# Prognostic factors of PSMA-targeted radioligand therapy in metastatic castration-resistant prostate cancer: a systematic review and meta-analysis

**DOI:** 10.1038/s41391-025-01034-y

**Published:** 2025-10-08

**Authors:** Takafumi Yanagisawa, Akihiro Matsukawa, Paweł Rajwa, Marcin Miszczyk, Tamás Fazekas, Benjamin Pradere, Keiichiro Miyajima, Yuki Enei, Angelo Cormio, Alessandro Dematteis, Timo Soeterik, Atsuya Okada, Hidetoshi Kuruma, Nat Lenzo, Shahrokh F. Shariat, Kenta Miki, Takahiro Kimura

**Affiliations:** 1 Department of Urology, Comprehensive Cancer Center, Medical University of Vienna, Vienna, Austria; 2 Department of Urology, The Jikei University School of Medicine, Tokyo, Japan; 3 Theranostics Yokohama, Yokohama, Japan; 4 Second Department of Urology, Centre of Postgraduate Medical Education, Warsaw, Poland; 5 Collegium Medicum - Faculty of Medicine, WSB University, Dąbrowa Górnicza, Poland; 6 Department of Urology, Semmelweis University, Budapest, Hungary; 7 Department of Urology, La Croix Du Sud Hospital, Quint Fonsegrives, France; 8 Department of Urology, Azienda Ospedaliero-Universitaria Ospedali Riuniti Di Ancona, Università Politecnica Delle Marche, Ancona, Italy; 9 Department of Surgical Sciences, Division of Urology, AOU Città della Salute e della Scienza at Molinette Hospital and University of Turin, Turin, Italy; 10 Department of Radiation Oncology, University Medical Center, Utrecht, Netherlands; 11 Jinsenkai MI Clinic, Osaka, Japan; 12 Bashamichi Sakura Clinic, Yokohama, Japan; 13 ICON Cancer Centre Theranostics, Perth, WA Australia; 14 Institute for Urology and Reproductive Health, Sechenov University, Moscow, Russia; 15 Division of Urology, Department of Special Surgery, The University of Jordan, Amman, Jordan; 16 Department of Urology, University of Texas Southwestern Medical Center, Dallas, TX USA; 17 Department of Urology, Second Faculty of Medicine, Charles University, Prague, Czech Republic; 18 Department of Urology, Weill Cornell Medical College, New York, NY USA; 19 Karl Landsteiner Institute of Urology and Andrology, Vienna, Austria; 20 Research Center for Evidence Medicine, Urology Department Tabriz University of Medical Sciences, Tabriz, Iran

**Keywords:** Outcomes research, Cancer therapy

## Abstract

**Background:**

Prostate-specific membrane antigen (PSMA)-targeted radioligand therapy (RLT) is a widely accepted treatment option for metastatic castration-resistant prostate cancer (mCRPC). However, synthesized evidence regarding potential prognostic factors for oncologic outcomes in patients treated with PSMA-RLT is lacking. We aimed to synthesize prognosticators of oncologic outcomes in patients with mCRPC treated with PSMA-RLT.

**Methods:**

PubMed®, Web of Science™, and Embase® databases were systemically searched in March 2025 for studies. Eligible studies investigated pretreatment clinical, hematologic, or radiographical prognostic factors for oncologic outcomes, such as progression-free (PFS) or overall survivals (OS) in patients with mCRPC treated with PSMA-RLT. Only parameters assessed through multivariable analysis adjusting for potential confounders were synthesized. (**CRD42024598718**)

**Results:**

A total of 39 studies (*n* = 4819) were included in the systematic review and 32 studies (*n* = 3038) were included in the meta-analysis. Prior chemotherapy (pooled HR: 1.43, 95%CI: 1.10–1.85), visceral metastases (pooled HR: 1.41, 95%CI: 1.05–1.89), and liver metastasis (pooled HR: 1.75, 95%CI: 1.37–2.25) were associated with worse PFS. Poor performance status (PS) (pooled HR: 1.99, 95%CI: 1.45–2.74), prior chemotherapy (pooled HR: 1.39, 95%CI: 1.19–1.63), visceral metastasis (pooled HR: 1.65, 95%CI: 1.33–2.05), bone metastasis (pooled HR: 2.09, 95%CI: 1.39–3.13), liver metastasis (pooled HR: 2.15, 95%CI: 1.84–2.50), and lower pretreatment hemoglobin levels (pooled HR: 1.25, 95%CI: 1.09–1.43) were associated with poorer OS. Higher pretreatment SUV_mean_ was associated with improved OS benefit (pooled HR: 0.91, 95%CI: 0.85–0.97). PSA decline after treatment initiation, particularly ≥50%, was associated with improved PFS and OS.

**Conclusions:**

Prior chemotherapy use and location of metastases influence the prognosis of patients with mCRPC treated with PSMA-RLT. A higher pre-treatment SUV_mean_ is predictive of better PSMA-RLT efficacy, and a greater PSA 'response is associated with improved survival outcomes. These findings may help guide clinical decision-making regarding PSMA-RLT and support prognostication of its oncological benefits.

## Introduction

Prostate-specific membrane antigen (PSMA) theranostics have transformed the landscape of diagnosis and treatment towards personalized medicine for prostate cancer (PCa), leveraging the high expression of PSMA on the membrane of PCa cells [1–5]. PSMA radioligand therapy (RLT), which combines PSMA-targeting ligands with radioactive isotopes, offers a highly selective and effective means of delivering localized radiation to tumor cells. This represents a personalized treatment approach for advanced PCa, particularly for metastatic castration-resistant prostate cancer (mCRPC) [1, 2].

PSMA-RLT utilizes small-molecule ligands conjugated with therapeutic radionuclides, most commonly Lutetium-177 (^177^Lu). Two randomized controlled trials (RCTs) have demonstrated a definitive survival benefit of ^177^Lu-PSMA-617 RLT compared to standard of care in patients with mCRPC who progressed following androgen receptor pathway inhibitors (ARPIs) or taxane-based chemotherapy [6–8]. To optimize the timing and selection of therapy- “the right treatment for the right patients at the right time” a nomogram incorporating several pretreatment clinical biomarkers was established in 2021 [9]. Notably, high PSMA expression levels on PSMA PET/CT have been considered a potential predictive marker for the efficacy of PSMA-RLT [10, 11].

However, despite well-established clinical and hematological prognostic factors for ARPIs and/or chemotherapy in mCRPC [12–14], robust evidence synthesizing potential predictive or prognostic factors specific to PSMA-RLT remains lacking. Such evidence is essential to support clinical decision-making. Therefore, this systematic review and meta-analysis aims to synthesize clinical, hematologic, and radiographical predictive and prognostic factors associated with oncologic outcomes in patients with mCRPC treated with PSMA-RLT.

## Materials and Methods

The protocol has been registered in the International Prospective Register of Systematic Reviews database (PROSPERO: **CRD42024598718**).

### Search strategy

This systematic review and meta-analysis was conducted based on the guidelines of the Preferred Reporting Items for Meta-Analyses of Observational Studies in Epidemiology Statement (Supplementary Table 2 [15]. In March 2025, a literature search on PubMed®, Web of Science™, and Embase® databases was performed to identify studies assessing the prognostic factors associated with oncologic outcomes of PSMA-RLT in mCRPC. The keywords used in our search strategy were as follows: “metastatic”, “advanced”, “prostate cancer”, “PSMA”, “survival”, and “progression”. The detailed database search strategy is shown in the Supplementary Appendix 1. The primary outcome of interest was overall survival (OS). Progression-free survival (PFS) was evaluated as a secondary endpoint. Two investigators performed initial screening based on the titles and abstracts to identify eligible studies. Potentially relevant studies were subjected to a full-text review. Additionally, manual search of references lists of relevant articles was also performed to identify additional studies. Disagreements were resolved by consensus with co-authors.

### Inclusion and exclusion criteria

Using the PECO framework [16], we defined our clinical question as follows: among patients with mCRPC treated with PSMA-RLT (Population), does the presence of specific prognostic factors (Exposure) compared to their absence (Comparator) influence OS and PFS (Outcome), as evaluated by multivariable Cox regression analysis in nonrandomized observational, randomized, or cohort studies (Study design). Studies lacking original patient data, reviews, letters, editorial comments, replies from authors, case reports, and articles not written in English were excluded. Additionally, studies not utilizing multivariable Cox regression analyses for OS and PFS were excluded. References of all papers included were scanned for additional studies of interest.

### Data extraction

Data were extracted independently by two authors. The first author’s name, publication year, recruitment country and periods, number of patients, age, performance status (PS), Gleason score (GS), prostate-specific antigen (PSA) at the initiation of PSMA-RLT, symptomatic disease, metastatic sites, number of prior treatment line and ARPIs, follow-up periods, median PFS and OS were extracted. Subsequently, the hazard ratios (HRs) and 95% confidence intervals (CIs) of pretreatment prognostic factors associated with PFS and OS were retrieved. All HRs were derived from multivariable analysis using Cox regression models. In cases of duplicate cohorts, the higher quality or the most recent data was extracted. All discrepancies were solved by consensus with co-authors.

### Risk of bias assessment

Assessment of study quality and risk of bias was carried out using the Risk-of-Bias version 2 (RoB2) tool [17] for randomized controlled trials (RCTs), and the Risk Of Bias In Non-randomized Studies of Interventions (ROBINS-I) tool version 2 for non-RCTs following the Cochrane Handbook for Systematic Reviews of Interventions [17]. For RoB2, each bias domain and overall risk of bias were judged as ‘Low’, ‘Some concerns’, or ‘High’ risk of bias (Supplementary Fig. 1). For ROBINS-I, ratings included ‘Low’, ‘Moderate’, ‘Serious’, or ‘Critical’ risk of bias (Supplementary Table 3). The main confounders were identified as the critical prognostic factors of OS. The presence of confounders was determined by consensus and review of the literature. The risk of bias assessment of each study was performed independently by two authors.

### Statistical analyses

Forest plots were used to analyze and summarize the multivariable HRs and describe the relationships between pretreatment clinical and hematologic factors and survival outcomes. Heterogeneity among the outcomes of included studies in this meta-analysis was assessed using Cochrane’s Q test and the I^2^ statistic. When significant heterogeneity was observed (*p*-value of <0.05 in the Cochrane Q test and a ratio >50% in I^2^ statistics), we attempted to identify its potential source. Given the diversity of included studies, a random-effects model was applied [18, 19]. Funnel plots were used for the assessment of publication bias (Supplementary Figs. 2 and 3). All statistical analyses were conducted using R version 4.3.0 (R Foundation for Statistical Computing, Vienna, Austria), and the statistical significance level was set at *P* < 0.05.

## Results

### Study selection and characteristics

**Fig. 1:**
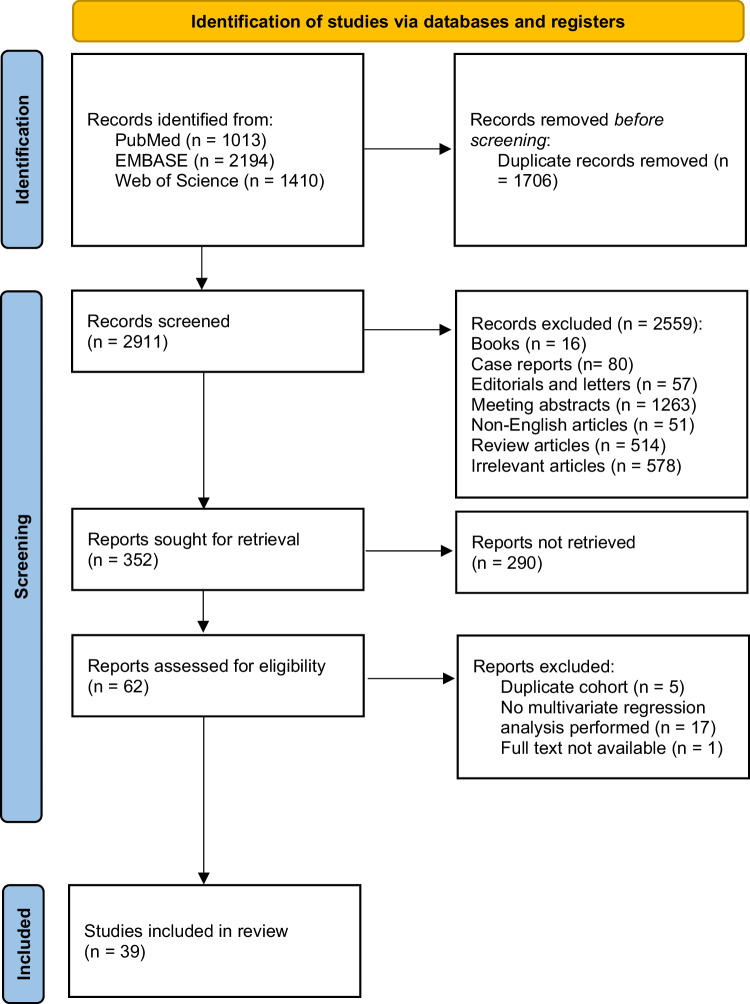
PRISMA 2020 flow diagram.

The literature search strategy is illustrated in Fig. 1. Following our inclusion and exclusion criteria, a total of 39 studies were included: two RCTs (*n* = 591) [11, 20, 21], five prospective cohort studies (*n* = 235) [22–26], and 32 retrospective cohort studies (*n* = 3993) [9, 27–59]. Details of patients’ baseline characteristics are presented in Table 1. Among the included studies, 35 studies evaluated oncologic outcomes in patients treated with 177Lu-PSMA-RLT [9, 11, 20–28, 30–51, 55–59], while two studies focused on 225Ac-PSMA-RLT [29, 52]. Additionally, two studies included various types of PSMA-RLT in their analysis, including 177Lu-, 225Ac-, and 90Y-PSMA RLT [53, 54].

**Table 1:** Characteristics of included studies.

Study /Author /Year	Study design	PSMA-targeted agent	Period	No. of Patients	Age (year)	ECOG PS	Follow-up, months	Gleason Score /ISUP grade group	Metastatic site
Slootbeek et al. 2024 [53]	RCS	177Lu-PSMA 225Ac-PSMA	2016–2023	95	61.8 (56.2–67.0)	NA	NA	GG1: 9 (9.5); GG2: 13 (13.7); GG3: 10 (10.5); GG4: 23 (24.2); GG5: 40 (42.1)	Bone: 85 (91.4) Visceral: 27 (29.0)
WARMTH Act Sathekge et al. 2024 [52]	RCS	225Ac-PSMA	2016–2023	488	68.1 (37–90)	0: 124 (25); 1: 194 (40); 2: 78 (16); 3: 42 (9); 4: 13 (3)	9.0 (IQR: 5.0–17.5)	GG1: 21 (6); GG2: 37 (10); GG3: 46 (13); GG4: 89 (25); GG5: 170 (47)	Bone: 435 (89) Lymph node: 352 (72) Visceral: 99 (20)
Satapathy et al. 2024[23]	PCS	177Lu-PSMA	2015–2023	40	68.5 (45–78)	0: 8 (20); 1: 3 (7.5); 2: 13 (32.5); 3: 13 (32.5); 4: 3 (7.5)	36 (95%CI: 26–46)	GS6: 0; GS7: 8 (20); GS8: 15 (37.5); GS9; 11 (27.5);GS10: 6 (15)	Bone: 39 (97.5) Lymph node: 21 (52.5) Visceral: 5 (12.5)
Raychaudhuri et al. 2024 [50]	RCS	177Lu-PSMA	NA	126	73 (68–78)	0: 46 (38); 1: 63 (52); 2: 12 (9.9): 3: 0; 4: 0	9.43 (95%CI: 8.77–10.5)	GG1-3: 26 (24); GG4–5: 83 (76)	Bone: 117 (93) Lymph node: 81 (64) Visceral: Liver: 20 (16); Lung: 20 (16)
Neubauer et al. 2024 [49]	RCS	177Lu-PSMA	2020–2022	73	75 (57–91)	NA	NA	GS: 8 (6–10)	Bone: 69 (95) Lymph node: 57 (78) Visceral: 18 (25)
Michalski et al. 2024 [47]	RCS	177Lu-PSMA	2018–2020	35	73.0 (46–90)	0–2: 35; 3–4: 0	NA	GS: 8 (5–10)	Bone: 35 (100) Lymph node: 17 (48.6) Visceral: Liver: 10 (28.6); Lung: 4 (11.4)
VISION Kuo et al. 2024 Armstrong et al. 2024 [11]	RCT	177Lu-PSMA	2018–2019	548	70.0 (64.0–75.0)	0–1: 508 (92.7);2: 40 (7.3); 3–4: 0	NA	NA	Bone: 501 (91.4) Lymph node: 273 (49.8) Visceral: Liver: 63 (11.5); Lung: 48 (8.8)
Kinikoglu et al. 2024 [46]	RCS	177Lu-PSMA	2017–2024	104	64.89 (44–85)	NA	NA	NA	NA
Kafka et al. 2024 [41]	RCS	177Lu-PSMA	2014–2022	233	64	0: 53; 1: 65; 2–3: 14; 4: 0	NA	GG1: 3; GG2-3: 27; GG4: 34; GG5: 64	NA
REALITY Hein et al. 2024 [38]	RCS	177Lu-PSMA	-2023	102	72 (48–88)	0: 29 (28.4); 1: 51 (50.0); 2–4: 22 (21.6)	44.4 (95%CI: 23.5–65.3)	NA	Bone: 93 (91.2) Lymph node: 79 (77.5) Visceral: 17 (16.7)
Hartrampf et al. 2024/2023 [37]	RCS	177Lu-PSMA	NA	73	72 (46–88)	NA	14	GS: 9 (6–10)	Bone: 72 (98.6) Lymph node: 49 (67.1) Visceral: 28 (38.4)
Eisazadeh et al. 2024 [34]	RCS	177Lu-PSMA	2018–2023	60	73 ± 8	NA	30 (3.0–37.1)	GG2: 5 (16); GG3-5: 14 (47)	NA
Wang et al.2023 [26]	PCS	177Lu-PSMA	2019–2021	30	68 (48–81)	0: 6 (20.0); 1: 16 (53.3); 2: 8 (26.7); 3–4: 0	23.8	GS: 8 (6–10)	Bone: 30 (100) Lymph node: 17 (56.7) Visceral: 9 (30)
Vanwelkenhuyzen et al. 2023 [25]	PCS	177Lu-PSMA	2020–2022	57	70.51 (64.88–74.85)	0–1: 41 (74.5); 2–4: 14 (25.5)	NA	GS5-7: 24 (42.1); GS8–10: 30 (52.6)	Bone: 52 (91.2) Lymph node: 37 (64.9) Visceral: 26 (45.6)
Thaiss et al. 2023 [56]	RCS	177Lu-PSMA	2017–2021	86	71 (52–95)	0: 36 (41.9); 1: 34 (39.5); 2: 14 (16.3); 3: 2 (2.3); 4: 0	12.4 (1–39)	GS6: 4 (4.7); GS7: 21 (24.4)GS8: 22 (25.6); GS): 30 (34.9);GS10: 2 (2.3)	Bone: 79 (91.9) Lymph node: 60 (70.0) Visceral: 39 (45.3)
Telli et al. 2023 [24]	PCS	177Lu-PSMA	2017–2019	52	70.3 ± 1.2	NA	17 (IQR: 10.2–20.7)	GS: 9 (5–10)	Bone: 33 (63.5) Lymph node: 7 (13.5) Visceral: 12 (23.1)
Tauber et al. 2023 [55]	RCS	177Lu-PSMA	2014–2022	80	82 (80–91)	0: 21 (26.6); 1: 51 (63.8); 2: 8 (10.0); 3–4: 0	11.4 (IWR: 5.8–16.7)	GS: 8 (5–10)	Bone: 73 (91.3) Visceral: 16 (20.0)
Stangl-Kremser et al. 2023 [54]	RCS	177Lu-J591 90Y-J591 177Lu-PSMA-617 225Ac-J591	2000–2021	180	71 (IQR: 66–77.5)	NA	18.8 (10.5–32.2)	GS: 8 (IQR: 7–9)	Bone: 167 (92.8) Lymph node: 141 (78.3) Visceral: Liver: 40 (22.2); Lung: 39 (21.7)
Şahin et al. 2023 [51]	RCS	177Lu-PSMA	2015–2023	61	69.8 ± 6.9	0–1: 33 (54); 2–3: 28 (46); 4: 0	53.2 ± 24	GS6-7: 22 (36); GS8-10: 39 (64)	Bone: 56 (92) Lymph node: 31 (51) Visceral: Lung: 3 (5); Liver: 3 (5); Other: 2 (3)
LuPIN Pathmanandavel et al. 2023 [22]	PCS	177Lu-PSMA	NA	56	68 (65–74)	0–1: 32 (86); 2: 5 (14); 3–4: 0	26	NA	NA
Kim et al. 2023 [44] Handke et al. 2023 [36]	RCS	177Lu-PSMA	2018–2020	78	71.2 ± 8.0	NA	NA	GS6: 5 (6.4); GS7: 10 (12.8);GS8: 30 (38.5); GS9: 24 (30.8); GS10:9 (11.5)	Bone: 70 (89.7) Lymph node: 49 (62.8) Visceral: Liver: 12 (15.4); Lung: 8 (10.3)
Karimzadeh et al. 2023 [42]	RCS	177Lu-PSMA	2014–2020	301	73 (66–77)	NA	9 (1–63)	NA	Bone: 274 Lymph node: 216 Visceral: 64
John et al. 2023 [40]	RCS	177Lu-PSMA	2019–2022	127	75 (70–80)	NA	NA	NA	Bone: 93 (97) Lymph node: 45 (47) Visceral: 19 (20)
Hotta et al. 2023 [39]	RCS	177Lu-PSMA	2014–2019	237	72 (IQR: 66–76)	NA	21.2 (IQR 14.1–30.6)	NA	Bone: 218 (92.0) Lymph node: 156 (65.8) Visceral: 72 (30.4)
Gaal et al. 2023 [35]	RCS	177Lu-PSMA	2015–2020	91	70 (65–76)	NA	19.8	NA	Bone: 87 (96) Lymph node: 79 (87) Visceral: Liver: 17 (19); Lung: 10 (11); Brain: 4 (4)
Cytawa et al. 2023 [33]	RCS	177Lu-PSMA	2015–2020	40	71.7 (54–91)	NA	17.3	NA	Bone: 36 (90.0) Lymph node: 31 (77.5) Visceral: 5 (12.5)
Chua et al. 2023 [32]	RCS	177Lu-PSMA	2018–2022	84	72.9 (68–77)	0: 29 (34.5); 1: 41 (48.8); 2: 14 (16.7); 3–4: 0	29.3 (IQR: 14.4–36.3)	GS: 9 (7–9)	Bone: 81 (96.4) Lymph node: 53 (63.1) Visceral: 21 (25.0)
Ballal et al. 2023 [29]	RCS	225Ac-PSMA	2018–2022	56	Refractory: 65 (61–70) Naïve: 70 (61.7–74)	Refractory: 3 (2–4) Naïve: 3 (2–4)	22 (95%CI: 18.3–24.8)	NA	Bone: 53 Lymph node: 53 Visceral: Lung: 11; Liver: 10; Brain: 3
Zarehparvar Moghadam et al. 2022 [59]	RCS	177Lu-PSMA	2016–2019	43	71 (51–88)	0–1: 23 (53.5); 2: 14 (32.6); 3–4: 6 (14)	17 (3–40)	GS: 9 (7–10)	Bone: 41 (95.3) Lymph node: 17 (39.5) Visceral: Liver: 6 (14); Lung: 5 (11.6)
Wrenger et al. 2022 [57]	RCS	177Lu-PSMA	2015–2020	52	72.1 (56.3–84.6)	NA	4–179 weeks	NA	Bone: 52 (100) Lymph node: 42 (80.8) Visceral: Liver: 9 (17.3); Lung: 3 (5.8); Brain: 3 (5.8)
Kemppainen et al. 2022 [43]	RCS	177Lu-PSMA	2017–2019	62	71.3 (IQR: 66.7–75.4)	NA	1.4 (IQR: 0.5–2.2) years	GS6: 8; GS7: 18; GS8: 15; GS9: 18; GS10: 1	Bone: 12 (19) Lymph node: 6 (10) Visceral: 14 (23)
Yadav et al. 2021 [58]	RCS	177Lu-PSMA	2014–2020	121	67 IQR: 60.7–72)	NA	16 (4–46)	GS6: 3 (2.4); GS7: 20 (16.5); GS8: 40 (33); GS9–10: 58 (48)	Bone: 78 (64) Lymph node: 82 (68) Visceral: Liver: 9 (7.5); Lung: 9 (7.5); Brain: 4 (3.3); Adrenal: 1 (0.8)
Michalski et al. 2021 [48]	RCS	177Lu-PSMA	2018–2020	54	71.4 (52–90)	0–3	NA	8 (5–10)	Bone: 52 Lumph node: 33 Visceral: Liver: 13; Lung: 8; Other: 9
Kind et al. 2021 [45]	RCS	177Lu-PSMA	2017–2019	27	73 (54–86)	NA	39.1 (95%CI: 32.1–46.1)	GS6: 0; GS7: 6 (22.2); GS8: 6 (22.2); GS9: 15 (55.6); GS10: 0	NA
Gafita et al. 2021 [9]	RCS	177Lu-PSMA	2014–2019	196	72 (67–76)	0–1: 173 (88); 2–4: 23 (12)	21.5 (IQR: 13.3–30.7)	NA	Bone: 179 (91) Lymph node: 129 (66) Visceral: Liver: 30 (15)
RESIST-PC Calais et al. 2021 [21]	RCT	177Lu-PSMA	2017–2018	43	74 (68–78)	0: 13 (30); 1: 21 (49); 2: 9 (21); 3–4: 0	24.8 (IQR: 22.9–28.8)	GG1-3: 18 (36); GG4-5: 25 (64)	Bone: 15 (35) Lymph node: 18 (42) Visceral: 15 (35)
WARMTH Ahmadzadehfar et al. 2021 [27]	RCS	177Lu-PSMA	2014–2018	416	71.9 (43–90)	0: 156 (37.5); 1: 166 (39.9); 2: 72 (17.3); 3–4: 0	NA	GS6-7: 114 (27.4); GS8-10: 239 (57.5)	Bone: 389 (92.8) Lymph node: 329 (79.1) Visceral: Liver: 87 (20.9); Lung: 68 (16.3); Brain: 10 (2.4)
Bülbül et al. 2020 [31]	RCS	177Lu-PSMA	2015–2018	45	64 (49–88)	NA	6 (1–32)	GS: 8 (6–10)	Bone: 39 (87) Lymph node: 30 (67) Visceral: Liver: 5 (11); Lung: 4 (9); Surrenal: 4 (9); Other: 3 (7)
Barber et al. 2019 [30]	RCS	177Lu-PSMA	2013–2016	167	Taxane pretreated: 69.3 ± 8.7 Taxane naïve: 70.8 ± 7.8	NA	10.3	Taxane pretreated GS6-7: 19 (23); GS8-10: 46 (55) Taxane naïve *177Lu* Lutetium-177, *225Ac* Actinium-225, *90Y* Yttrium-90, *CI* Confidence Interval, *ECOG PS* Eastern Cooperative Oncology Group Performance Status, *GG* Grade Group, *GS* Gleason Score, *ISUP* International Society of Urological Pathology, *IQR* Interquartile Range, *J591* Monoclonal Antibody Targeting PSMA, *NA* Not Available, *PCS* Prospective Cohort Study, *PSMA* Prostate-Specific Membrane Antigen, *RCS* Retrospective Cohort Study, *RCT* Randomized Controlled Trial	Bone: 141 (84.4) Lymph node: 136 (81.4) Visceral: 36 (21.6)

*177Lu* Lutetium-177, *225Ac* Actinium-225, *90Y* Yttrium-90, *CI* Confidence Interval, *ECOG PS* Eastern Cooperative Oncology Group Performance Status, *GG* Grade Group, *GS* Gleason Score, *ISUP* International Society of Urological Pathology, *IQR* Interquartile Range, *J591* Monoclonal Antibody Targeting PSMA, *NA* Not Available, *PCS* Prospective Cohort Study, *PSMA* Prostate-Specific Membrane Antigen, *RCS* Retrospective Cohort Study, *RCT* Randomized Controlled Trial.

### Assessment of risk of bias and quality of study

Risk of bias assessment for each study is summarized in Supplementary Fig. 1 and Supplementary Table 3. According to the RoB2 tool for RCTs, two were classified as having some concerns regarding bias. According to the ROBINS-I tool for non-RCTs, 35 studies were classified as having a moderate risk, and two studies as having a low risk of bias.

### Meta-analyses

**Fig. 2:**
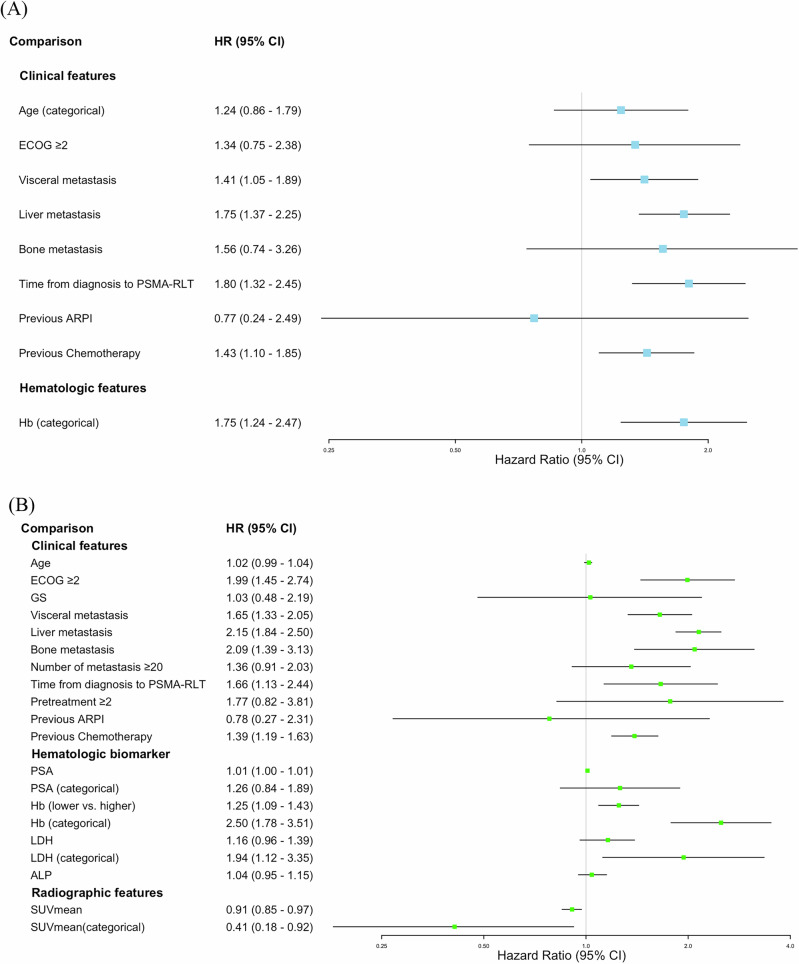
Forest plots of meta-analysis of prognostic factors for survival outcomes. **A** OS and **B** PFS.

A total of 32 studies, comprising 3038 patients with mCRPC who underwent PSMA-RLT, were included in the meta-analyses. Although the studies by Michalski et al. [48], Hartrampf et al. [37], and Cytawa et al. [33] were conducted at the same institutions and appeared to involve potentially overlapping cohorts, they were considered distinct based on differences in inclusion criteria and reported outcomes. Therefore, all were included in the present study. The same rationale was applied to the studies by Tauber et al. [55] and Karimzadeh et al. [42]. Figure 2 depicts forest plots of prognostic factors for PFS (Fig. 2A) and OS (Fig. 2B).

#### Pretreatment clinical features

As shown in Fig. 2, Age (pooled HR: 1.24, 95% CI: 0.86–1.79, *p* = 0.2) and poor Eastern Cooperative Oncology Group (ECOG) PS (pooled HR: 1.34, 95% CI: 0.75–2.38, *p* = 0.3) were not significantly associated with PFS. In contrast, presence of visceral metastases (pooled HR: 1.41, 95% CI: 1.05–1.89, *p* = 0.02) and liver metastases (pooled HR: 1.75, 95% CI: 1.37–2.25, *p* < 0.001) were significantly associated with worse PFS, whereas bone metastases were not (pooled HR: 1.56, 95% CI: 0.74–3.26, *p* = 0.2). A longer time from diagnosis to PSMA-RLT was also significantly associated with worse PFS (pooled HR: 1.80, 95% CI: 1.32–2.45, *p* < 0.001). Previous chemotherapy use was associated with a significantly increased risk of progression compared to no previous chemotherapy (pooled HR: 1.43, 95% CI: 1.10–1.85, *p* < 0.01), whereas previous ARPIs use showed no significant association (pooled HR: 0.77, 95% CI: 0.24–2.49, *p* = 0.7). Although significant heterogeneity was observed in the analysis of previous ARPI use (I^2^ = 81.4%, Cochran’s Q test = 0.4, *p* = 0.02), only two studies were included, limiting the ability to identify the source of heterogeneity.

Regarding OS, Poor ECOG PS was significantly associated with worse OS (pooled HR: 1.99, 95% CI: 1.45–2.74, *p* < 0.001), whereas age (pooled HR: 1.02, 95% CI: 0.99–1.04, *p* = 0.2) and GS (pooled HR: 1.03, 95% CI: 0.48–2.19, *p* > 0.9) were not. Significant association with worse OS were also observed for visceral metastases (pooled HR: 1.65, 95% CI: 1.33–2.05, *p* < 0.001), liver metastases (pooled HR: 2.15, 95% CI: 1.83–2.56, *p* < 0.001), and bone metastases (pooled HR: 2.09, 95% CI: 1.39–3.13, *p* < 0.001). Similarly, a longer time from diagnosis to PSMA-RLT was significantly associated with worse OS (pooled HR: 1.66, 95% CI: 1.13–2.44, *p* = 0.01). Previous chemotherapy use was also associated with significantly worse OS compared to no previous chemotherapy (pooled HR: 1.39, 95% CI: 1.19–1.63, *p* < 0.001), whereas previous ARPIs use showed no significant association (pooled HR: 0.78, 95% CI: 0.27–2.32, *p* = 0.7). Although significant heterogeneity was observed in the analysis of GS, only two studies were included, limiting our ability to explore the source of heterogeneity.

#### Pretreatment Hematologic Biomarkers

Lower pretreatment Hb levels were significantly associated with worse PFS (pooled HR: 1.75, 95% CI: 1.24–2.47, p < 0.01, Fig. 2A) and worse OS (pooled HR: 1.25, 95% CI: 1.09–1.43, p < 0.01, Fig. 2B). No significant associations with OS were observed for pretreatment PSA levels (pooled HR: 1.01, 95% CI: 1.00–1.01, p = 0.3), LDH (pooled HR: 1.16, 95% CI: 0.96–1.39, p = 0.1), or ALP (pooled HR: 1.04, 95% CI: 0.95–1.15, p = 0.4). Significant heterogeneity was observed in the analyses of PSA, Hb, LDH, and ALP for OS. For PSA and LDH. All included studies were retrospective, and subgroup analyses by study design could not be conducted. Leave-one-out analyses did not identify any single study as a clear source of heterogeneity. In contrast, the heterogeneity observed for Hb appeared to be primarily driven by the study by Telli et al. [24], which was the only prospective study among those included. Similarly, for ALP, the VISION trial-an RCT-was identified as a potential source of heterogeneity based on the leave-one-out analysis [11, 20].

#### Pretreatment Radiographic Features

Higher SUVmean was significantly associated with longer OS (pooled HR: 0.91, 95% CI: 0.85–0.97, p < 0.01). Although significant heterogeneity was observed (I^2^ = 79.6%, Cochran’s Q test = 4.9, p = 0.03), the source of heterogeneity could not be identified due to the limited number of included studies (n = 2).

#### PSA kinetics

**Fig. 3:**
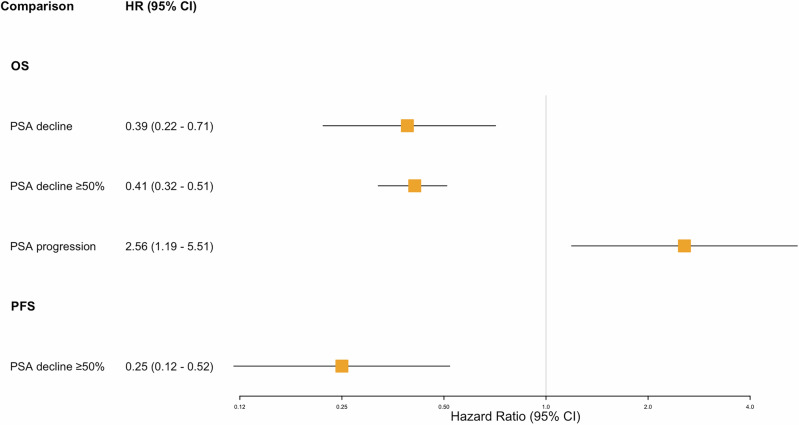
Forest plots of meta-analysis of oncological outcomes based on PSA kinetics.

A PSA decline of more than 50% was significantly associated with improved PFS (pooled HR: 0.25, 95% CI: 0.12–0.52, p < 0.001) and OS (pooled HR: 0.41, 95% CI: 0.32–0.51, p < 0.001) (Fig. 3). Conversely, PSA progression was significantly associated with worse OS (pooled HR: 2.56, 95% CI: 1.19–5.51, p = 0.02). Significant heterogeneity was observed in the analysis of PSA decline ≥50% for PFS (I^2^ = 80.9%, Cochran’s Q test = 10.5, p < 0.01). Leave-one-out analysis suggests that the study by Sathekge [52] might be the primary source of this heterogeneity.

## Discussion

This is the first meta-analysis comprehensively assessing clinical, hematological, and radiographical prognostic factors for oncologic outcomes in patients with mCRPC treated with PSMA-RLT. Although the European Association of Nuclear Medicine (EANM) guidelines summarize several clinical, radiological, and laboratory prognostic factors associated with survival outcomes [60], the supporting evidence remains limited. We identified several key findings. First, regarding clinical settings and features, prior chemotherapy, the presence of visceral metastases (particularly liver metastases), and poor PS were consistent prognostic factors associated with worse outcomes, despite PSMA-RLT. Second, among hematologic parameters, only low pretreatment Hb was a reliable predictor of poorer oncologic outcomes. Third, with respect to radiographic features, high pretreatment SUVmean values were associated with improved OS. Finally, post-treatment PSA kinetics, particularly achieving a ≥ 50% decline in PSA, was strongly associated with better long-term oncologic outcomes following PSMA-RLT.

Given that ARPIs are now the mainstay of treatment for metastatic PCa, chemotherapy status substantially affects disease biology and prognosis. Chemotherapy is typically administered in later-line settings following progression after ARPI treatment. Since two RCTs demonstrated the survival benefit of 177-Lu-PSMA-617 in post-docetaxel mCRPC patients in 2021, PSMA-RLT has been widely used in this population [6, 7]. More recently, the PSMAfore trial demonstrated a radiographical PFS benefit of 177-Lu-PSMA-617 over ARPI switching in chemo-naïve mCRPC [61]. Although no OS benefit was shown-likely due to high crossovers to PSMA-RLT in the control arm- this study has driven a shift toward earlier use of PSMA-RLT [61, 62]. Furthermore, the ENZA-p trial demonstrated that combining 177Lu-PSMA RLT with enzalutamide yielded superior PFS compared to enzalutamide alone in the chemo-naïve mCRPC setting [63]. Collectively, our analysis supports that prior chemotherapy is a poor prognostic indicator and reinforces the value of earlier PSMA-RLT administration, potentially in combination with ARPIs.

We further identified several clinical prognostic factors for poor oncologic outcomes in patients with mCRPC treated with PSMA-RLT. The negative prognostic effect of visceral metastases and poor PS are consistent with prior meta-analysis in patients with post-docetaxel mCRPC [14]. Visceral metastasis, particularly liver involvement, are widely recognized as markers of aggressive disease and are included in the definition of high-volume disease in metastatic hormone-sensitive PCa [64, 65]. While novel systemic therapies, including ARPIs and chemotherapy, show efficacy regardless of visceral metastases [66], liver metastasis in particular remains strong negative prognostic factor. Subgroup analysis from the VISION trial revealed that although the PFS benefit of 177Lu-PSMA RLT was comparable between patients with and without liver metastasis (HR: 0.28 vs. 0.43), the OS benefit was attenuated in those with liver metastasis (HR: 0.87 vs. 0.62) [7]. Our analysis identified liver metastasis as the strongest prognostic factors of poor OS (HR: 2.15, 95%CI: 1.84–2.50), underscoring its prognostic impact and importance in shared-decision making.

Among hematologic biomarkers, commonly used markers such as pretreatment PSA, ALP, and LDH showed limited prognostic value in the context of PSMA-RLT. Only low pretreatment Hb was consistently associated with poor OS and PFS, aligning with the nomogram proposed by Gafita A et al. in 2021 [9]. One possible explanation may be that severe anemia ( ≥ CTCAE grade3) is a common adverse event of 177Lu-PSMA RLT, reported in 8–13% of patients in the TheraP and VISION trials [6, 7]. This may be in part related to heavy pre-treatment, particularly previous large field radiotherapy, and large volume of osseous disease in the patients going onto 177Lu-PSMA RLT. Therefore, clinicians should carefully assess eligibility of PSMA-RLT in patients with prior anemia. Other possible inflammation-related or nutritional markers, which may reflect cancer cachexia or sarcopenia, have also shown prognostic utility in mCRPC [14, 67]. For example, sarcopenia was recently identified as a strong prognostic factor in patients treated with cabazitaxel [68]. While we did not include these parameters, prior studies have demonstrated the potential of markers, such as neutrophil-lymphocyte ratio, systemic inflammation index, and De Ritis ratio [35, 51]. Future research should investigate these accessible and clinically relevant biomarkers further.

High PSMA expression levels on PET/CT are regarded as a prerequisite for effective PSMA-RLT [2]. Post hoc analyses from major RCTs confirmed the predictive value of SUVmean on PSMA PET/CT [9, 10]. In the TheraP trial, patients with a whole-body SUV mean≥10 showed superior rPFS and PSA-PFS compared to those with lower values [10]. Similarly, the VISION trial confirmed that SUV mean stratified both rPFS and OS [11]. Our findings corroborate this, reinforcing the importance of SUVmean as a prognostic and predictive biomarker. While many current methods rely on manual or semi-automatic delineation of regions of interest (ROIs), these approaches are subject to inter-observer variability and may lack reproducibility. In this context, emerging artificial intelligence (AI)-assisted diagnostic tools may enhance evaluation of PSMA PET/CT metrics, offering more refined assessment of disease burden and prognosis [69].

Despite widespread use of radiographical assessment for the treatment efficacy of mPCa, PSA kinetics remains a clinically meaningful prognostic tool for mCRPC and mHSPC patients [70, 71]. Our findings reaffirmed that a ≥ 50% PSA decline correlates with improved OS and PFS in patients treated with the PSMA-RLT, in agreement with prior meta-analysis [72]. While standardized imaging criteria for assessing PSMA-RLT efficacy are still lacking, PSA kinetics provides a practical surrogate. Future studies should explore AI-based imaging analysis (e.g., changes in SUV mean) to further refine treatment evaluation [69].

This study has several limitations. First, most included studies were retrospective, introducing potential selection bias. Second, unknown pretreatment factors (e.g., nutritional status, comorbidities, medications, lifestyle) may have influenced clinical and hematologic biomarkers, contributing to systematic bias. Third, cutoff values for hematologic markers were inconsistent and determined using heterogeneous statistical methods or predefined ranges from prior literature. Fourth, despite using a random-effects model to address study heterogeneity, results should be interpreted with caution. Treatment regimens, including radioligand type, administered dose, and number of cycles, varied across studies. Fifth, these findings were generated mainly from Lutetium-177-based PSMA targeting agents. The increased binding of PSMA binding antibodies and the higher linear energy transfer of radio-isotopes such as Acinium-255 and Lead-212 may mean that certain factors such as SUV cut off will likely be altered in situations using these new types of PSMA targeting theranostic agent. Lastly, genomic data were not analyzed, although emerging evidence suggests certain genomic alterations may mediate resistance to PSMA-RLT despite high PSMA expression [50, 73, 74]. Future incorporation of genomic profiling, such as circulating tumor DNA, will be critical for advancing personalized therapy in this context [75].

## Conclusions

Prior chemotherapy was associated with poorer prognosis in mCRPC patients receiving PSMA-RLT. Visceral metastases, particularly liver metastases, were strong negative prognostic indicators. Patients with low pretreatment hemoglobin require careful consideration due to the risk of hematologic toxicity. In contrast, higher SUVmean values were associated with better OS, supporting their predictive and prognostic value. A robust PSA response was also linked to improved survival. These findings may inform patient selection, risk stratification, and shared decision-making regarding PSMA-RLT. Nonetheless, further investigation is needed into the prognostic implications of sarcopenia, AI-assisted radiographic assessment, and genomic biomarkers to optimize candidate selection for PSMA-RLT.

## Supplementary information

Supplementary Information
