# Supplementary material for: Prognostic factors of PSMA-targeted radioligand therapy in metastatic castration-resistant prostate cancer: a systematic review and meta-analysis
Source: Prostate Cancer Prostatic Dis. 2025 Oct 8;29(2):258–70. doi: 10.1038/s41391-025-01034-y (PMC13190232; doi:10.1038/s41391-025-01034-y)

**Supplementary Information**

**Supplementary Table 1.** PRISMA 2020 for Abstracts Checklist

**Supplementary Table 2.** PRISMA 2020

**Supplementary Table 3.** ROBINS-I version.2

**Supplementary Table 4.** Summary of prognostic factors for OS in each included study

**Supplementary Table 5.** Summary of prognostic factors for PFS in each included study

**Supplementary Table 6.** Summary of oncological outcomes based on PSA kinetics

**Supplementary Table 7.** Summary of meta-analyses for OS

**Supplementary Table 8.** Summary of meta-analyses for PFS

**Supplementary Table 9.**Summary of leave-one-out analysis

**Supplementary Figure 1.** RoB2

**Supplementary Figure 2.** Funnel plots of meta-analysis for OS

**Supplementary Figure 3.** Funnel plots of meta-analysis for PFS

**Supplementary Appendix 1**. Search strategy for systematic review and meta-analysis

**Supplementary Appendix 2**. AMSTAR-2 checklist

**Abbreviations:**

AE = Adverse Events, AMSTAR-2 = A Measurement Tool to Assess Systematic Reviews, PRISMA = Preferred Reporting Items for Systematic Reviews and Meta-Analyses, RoB2 = Risk of Bias 2, ROBINS-I = Risk of Bias In Non-randomized Studies of Interventions

**Supplementary Table 1.** PRISMA 2020 for Abstracts Checklist

| **Section and Topic** | **Item #** | **Checklist item** | **Reported (Yes/No)** |
| --- | --- | --- | --- |
| **TITLE** | | |  |
| Title | 1 | Identify the report as a systematic review. | Yes |
| **BACKGROUND** | | |  |
| Objectives | 2 | Provide an explicit statement of the main objective(s) or question(s) the review addresses. | Yes |
| **METHODS** | | |  |
| Eligibility criteria | 3 | Specify the inclusion and exclusion criteria for the review. | Yes |
| Information sources | 4 | Specify the information sources (e.g. databases, registers) used to identify studies and the date when each was last searched. | Yes |
| Risk of bias | 5 | Specify the methods used to assess risk of bias in the included studies. | No |
| Synthesis of results | 6 | Specify the methods used to present and synthesise results. | Yes |
| **RESULTS** | | |  |
| Included studies | 7 | Give the total number of included studies and participants and summarise relevant characteristics of studies. | Yes |
| Synthesis of results | 8 | Present results for main outcomes, preferably indicating the number of included studies and participants for each. If meta-analysis was done, report the summary estimate and confidence/credible interval. If comparing groups, indicate the direction of the effect (i.e. which group is favoured). | Yes |
| **DISCUSSION** | | |  |
| Limitations of evidence | 9 | Provide a brief summary of the limitations of the evidence included in the review (e.g. study risk of bias, inconsistency and imprecision). | No |
| Interpretation | 10 | Provide a general interpretation of the results and important implications. | Yes |
| **OTHER** | | |  |
| Funding | 11 | Specify the primary source of funding for the review. | Yes |
| Registration | 12 | Provide the register name and registration number. | No |

**Supplementary Table 2.** PRISMA 2020

| **Section and Topic** | **Item #** | **Checklist item** | **Location where item is reported** |
| --- | --- | --- | --- |
| **TITLE** | | |  |
| Title | 1 | Identify the report as a systematic review. | P1 |
| **ABSTRACT** | | |  |
| Abstract | 2 | See the PRISMA 2020 for Abstracts checklist. | P3 |
| **INTRODUCTION** | | |  |
| Rationale | 3 | Describe the rationale for the review in the context of existing knowledge. | P4 |
| Objectives | 4 | Provide an explicit statement of the objective(s) or question(s) the review addresses. | P4 |
| **METHODS** | | |  |
| Eligibility criteria | 5 | Specify the inclusion and exclusion criteria for the review and how studies were grouped for the syntheses. | P5-7 |
| Information sources | 6 | Specify all databases, registers, websites, organisations, reference lists and other sources searched or consulted to identify studies. Specify the date when each source was last searched or consulted. | P5-7 |
| Search strategy | 7 | Present the full search strategies for all databases, registers and websites, including any filters and limits used. | P5-7 |
| Selection process | 8 | Specify the methods used to decide whether a study met the inclusion criteria of the review, including how many reviewers screened each record and each report retrieved, whether they worked independently, and if applicable, details of automation tools used in the process. | P5-7 |
| Data collection process | 9 | Specify the methods used to collect data from reports, including how many reviewers collected data from each report, whether they worked independently, any processes for obtaining or confirming data from study investigators, and if applicable, details of automation tools used in the process. | P5-7 |
| Data items | 10a | List and define all outcomes for which data were sought. Specify whether all results that were compatible with each outcome domain in each study were sought (e.g. for all measures, time points, analyses), and if not, the methods used to decide which results to collect. | P5-7 |
|  | 10b | List and define all other variables for which data were sought (e.g. participant and intervention characteristics, funding sources). Describe any assumptions made about any missing or unclear information. | P5-7 |
| Study risk of bias assessment | 11 | Specify the methods used to assess risk of bias in the included studies, including details of the tool(s) used, how many reviewers assessed each study and whether they worked independently, and if applicable, details of automation tools used in the process. | P5-7 |
| Effect measures | 12 | Specify for each outcome the effect measure(s) (e.g. risk ratio, mean difference) used in the synthesis or presentation of results. | P5-7 |
| Synthesis methods | 13a | Describe the processes used to decide which studies were eligible for each synthesis (e.g. tabulating the study intervention characteristics and comparing against the planned groups for each synthesis (item #5)). | P5-7 |
|  | 13b | Describe any methods required to prepare the data for presentation or synthesis, such as handling of missing summary statistics, or data conversions. | P5-7 |
|  | 13c | Describe any methods used to tabulate or visually display results of individual studies and syntheses. | P5-7 |
|  | 13d | Describe any methods used to synthesize results and provide a rationale for the choice(s). If meta-analysis was performed, describe the model(s), method(s) to identify the presence and extent of statistical heterogeneity, and software package(s) used. | P5-7 |
|  | 13e | Describe any methods used to explore possible causes of heterogeneity among study results (e.g. subgroup analysis, meta-regression). | P5-7 |
|  | 13f | Describe any sensitivity analyses conducted to assess robustness of the synthesized results. | P5-7 |
| Reporting bias assessment | 14 | Describe any methods used to assess risk of bias due to missing results in a synthesis (arising from reporting biases). | P5-7 |
| Certainty assessment | 15 | Describe any methods used to assess certainty (or confidence) in the body of evidence for an outcome. | P5-7 |
| **RESULTS** | | |  |
| Study selection | 16a | Describe the results of the search and selection process, from the number of records identified in the search to the number of studies included in the review, ideally using a flow diagram. | P8-10 |
|  | 16b | Cite studies that might appear to meet the inclusion criteria, but which were excluded, and explain why they were excluded. | P8-10 |
| Study characteristics | 17 | Cite each included study and present its characteristics. | P8-10 |
| Risk of bias in studies | 18 | Present assessments of risk of bias for each included study. | P8-10 |
| Results of individual studies | 19 | For all outcomes, present, for each study: (a) summary statistics for each group (where appropriate) and (b) an effect estimate and its precision (e.g. confidence/credible interval), ideally using structured tables or plots. | P8-10 |
| Results of syntheses | 20a | For each synthesis, briefly summarise the characteristics and risk of bias among contributing studies. | P8-10 |
|  | 20b | Present results of all statistical syntheses conducted. If meta-analysis was done, present for each the summary estimate and its precision (e.g. confidence/credible interval) and measures of statistical heterogeneity. If comparing groups, describe the direction of the effect. | P8-10 |
|  | 20c | Present results of all investigations of possible causes of heterogeneity among study results. | P8-10 |
|  | 20d | Present results of all sensitivity analyses conducted to assess the robustness of the synthesized results. | P8-10 |
| Reporting biases | 21 | Present assessments of risk of bias due to missing results (arising from reporting biases) for each synthesis assessed. | P8-10 |
| Certainty of evidence | 22 | Present assessments of certainty (or confidence) in the body of evidence for each outcome assessed. | P8-10 |
| **DISCUSSION** | | |  |
| Discussion | 23a | Provide a general interpretation of the results in the context of other evidence. | P11-13 |
|  | 23b | Discuss any limitations of the evidence included in the review. | P11-13 |
|  | 23c | Discuss any limitations of the review processes used. | P11-13 |
|  | 23d | Discuss implications of the results for practice, policy, and future research. | P11-13 |
| **OTHER INFORMATION** | | |  |
| Registration and protocol | 24a | Provide registration information for the review, including register name and registration number, or state that the review was not registered. | P5 |
|  | 24b | Indicate where the review protocol can be accessed, or state that a protocol was not prepared. | P5 |
|  | 24c | Describe and explain any amendments to information provided at registration or in the protocol. | P5 |
| Support | 25 | Describe sources of financial or non-financial support for the review, and the role of the funders or sponsors in the review. | P17 |
| Competing interests | 26 | Declare any competing interests of review authors. | P17 |
| Availability of data, code and other materials | 27 | Report which of the following are publicly available and where they can be found: template data collection forms; data extracted from included studies; data used for all analyses; analytic code; any other materials used in the review. | NA |

**Supplementary Table 3.** ROBINS-I version.2

| Study | D1 | D2 | D3 | D4 | D5 | D6 | D7 | Overall |
| --- | --- | --- | --- | --- | --- | --- | --- | --- |
| Slootbeek et al. 2024 | Moderate | Low | Low | Low | Moderate | Low | Low | Moderate |
| WARMTH Act Sathekge et al. 2024 | Low | Low | Low | Low | Moderate | Low | Low | Moderate |
| Satapathy et al. 2024 | Low | Low | Moderate | Low | Low | Low | Low | Moderate |
| Raychaudhuri et al. 2024 | Low | Low | Moderate | Low | Low | Low | Moderate | Moderate |
| Neubauer et al. 2024 | Low | Low | Low | Low | Low | Low | Moderate | Moderate |
| Michalski et al. 2024 | Low | Low | Moderate | Low | Low | Low | Moderate | Moderate |
| Kinikoglu et al. 2024 | Low | Moderate | Low | Low | Low | Low | Low | Moderate |
| Kafka et al. 2024 | Low | Low | Low | Low | Low | Low | Moderate | Moderate |
| REALITY Hein et al. 2024 | Low | Low | Low | Low | Low | Low | Moderate | Moderate |
| Hartrampf et al. 2024/2023 | Low | Low | Low | Low | Low | Low | Moderate | Moderate |
| Eisazadeh et al. 2024 | Low | Low | Low | Low | Low | Moderate | Low | Moderate |
| Wang et al.2023 | Low | Low | Moderate | Low | Low | Moderate | Low | Moderate |
| Vanwelkenhuyzen et al. 2023 | Low | Low | Moderate | Low | Low | Moderate | Low | Moderate |
| Thaiss et al. 2023 | Low | Low | Low | Low | Low | Moderate | Low | Moderate |
| Telli et al. 2023 | Low | Low | Moderate | Low | Low | Low | Low | Moderate |
| Tauber et al. 2023 | Moderate | Low | Low | Low | Low | Low | Low | Moderate |
| Stangl-Kremser et al. 2023 | Low | Low | Low | Low | Low | Moderate | Low | Moderate |
| Şahin et al. 2023 | Moderate | Low | Low | Low | Low | Moderate | Low | Moderate |
| LuPIN Pathmanandavel et al. 2023 | Low | Low | Low | Low | Low | Moderate | Low | Moderate |
| Kim et al.  2023 | Low | Low | Moderate | Low | Low | Low | Moderate | Moderate |
| Karimzadeh et al. 2023 | Low | Low | Moderate | Low | Low | Low | Low | Moderate |
| John et al. 2023 | Low | Low | Moderate | Low | Low | Low | Low | Moderate |
| Hotta et al. 2023 | Low | Low | Low | Low | Low | Low | Moderate | Moderate |
| Gaal et al. 2023 | Low | Low | Moderate | Low | Low | Low | Low | Moderate |
| Cytawa et al. 2023 | Moderate | Low | Low | Low | Low | Low | Low | Moderate |
| Chua et al. 2023 | Moderate | Low | Low | Low | Low | Low | Low | Moderate |
| Ballal et al. 2023 | Moderate | Low | Moderate | Low | Low | Low | Low | Moderate |
| Zarehparvar Moghadam et al. 2022 | Moderate | Low | Low | Low | Low | Low | Low | Moderate |
| Wrenger et al. 2022 | Moderate | Low | Moderate | Low | Low | Low | Low | Moderate |
| Kemppainen et al. 2022 | Moderate | Low | Low | Low | Low | Low | Low | Moderate |
| Yadav et al. 2021 | Moderate | Low | Low | Low | Low | Low | Low | Moderate |
| Michalski et al. 2021 | Moderate | Moderate | Low | Low | Low | Low | Low | Moderate |
| Kind et al. 2021 | Low | Low | Low | Low | Low | Low | Moderate | Moderate |
| Gafita et al. 2021 | Low | Low | Low | Low | Low | Low | Low | Low |
| WARMTH Ahmadzadehfar et al. 2021 | Low | Low | Low | Low | Low | Low | Low | Low |
| Bülbül et al. 2020 | Moderate | Low | Low | Low | Low | Low | Low | Moderate |
| Barber et al. 2019 | Low | Moderate | Low | Low | Low | Low | Low | Moderate |

D1: Risk of bias due to confounding

D2: Risk of bias in classification of interventions

D3: Risk of bias in selection of participants into the study (or into the analysis)

D4: Risk of bias due to deviations from intended interventions

D5: Risk of bias due to missing data

D6: Risk of bias arising from measurement of the outcome

D7: Risk of bias in selection of the reported results

**Supplementary Table 4.** Summary of prognostic factors for OS in each included study

|  | Outcomes HR (95% CI) | | | | | |
| --- | --- | --- | --- | --- | --- | --- |
| Study | Clinical features | HR (95% CI) | Hematologic biomarkers | HR (95% CI) | Radiographic features | HR (95% CI) |
| Slootbeek et al. (2024) | Gene mutation  TP53  AR  MYC  PI3K  BRCA1/2  HRR  MAPK | : 2.53 (1.52-4.22) : 0.85 (0.69-1.05) : 0.61 (0.26-1.45) : 1.13 (0.7-1.82) : 0.9 (0.43-1.89) : 1.07 (0.61-1.88) : 0.99 (0.33-3.02) | NA |  | NA |  |
| Sathekge et al. (2024) | Previous chemotherapy Previous ARPI Treatment with 177Lu-PSMA RLT Liver metastasis Peritoneal metastasis Number of cycles of 225Ac-PSMA RLT | : 1.114 (0.77-1.61) : 1.175 (0.82-1.68) : 1.213 (0.88-1.68)  : 1.895 (1.25-2.87) : 5.025 (2.18-11.60) : 0.924 (0.85-1.01) | Anemia | : 1.615 (1.198-2.176) | NA |  |
| Satapathy et al. (2024) | Number of bone metastasis ≥20 Visceral metastasis | : 2.78 (1.11-6.93)  : 1.74 (0.54-5.58) | NA |  | NA |  |
| Raychaudhuri et al. (2024) | Liver metastasis PARPi received Gene mutation  DDR  TSG | : 3.19 (1.18-8.6) : 1.81 (0.66-4.94)  : 0.37 (0.14-0.97) : 2.65 (1.15-6.11) | NA |  | PSMA SUVmean≥10 | : 0.4 (0.09-1.71) |
| Neubauer et al. (2024) | Age GS | : 0.99 (0.94-1.04) : 1.51 (0.99-2.34) | ALP LDH | : 0.74 (0.27-1.97) : 4.54 (1.77-12.08) | TTV decrease | : 0.32 (0.13-0.76) |
| Michalski et al. (2024) | PSA GS Previous CBZ VISION criteria | : 1.00 (1.00-1.00) : 0.70 (0.45-1.09) : 1.18 (0.45-2.81) : 0.28 (0.12-0.71) | NA |  | NA |  |
| Kuo et al. (2024) Armstrong et al. (2024) | Bone metastasis Liver metastasis Lymph node metastasis Age ECOG PS≥2 | : 2.78 (1.52-5.26) : 1.75 (1.25-2.44) : 0.69 (0.53-0.89) : 1.02 (1.00-1.03) : 2.25 (1.50-3.37) | PSA ALP LDH | : 1.12 (1.01-1.26) : 1.19 (1.06-1.33) : 1.42 (1.23-1.64) | SUVmean SUVmean(Bone) SUVmean(Lymoh node) SUVmax(Soft tissue) | : 0.88 (0.84-0.91) : 0.89 (0.85-0.93) : 0.93 (0.90-0.96) : 0.99 (0.98-1.00) |
| Kinikoglu et al. (2024) | Age(≥65) Gleason Group (High/very high vs. Intermediate) Visceral metastasis Previous chemotherapy 177-Lu line (≥3 vs. 2) Previous ARPI Combination therapy | : 2.70 (1.49-4.76) : 1.22 (0.64-2.33)  : 1.61 (0.94-2.70) : 1.11 (0.48-2.56) : 0.93 (0.32-2.66) : 0.37 (0.10-1.33) : 0.35 (0.19-0.67) | NA |  | NA |  |
| Kafka et al. (2024) | NA |  | NA |  | NA |  |
| Hein et al. (2024) | NA |  | Hb | : 0.90 (0.78-1.04) | TLP MTV | : 1.03 (1.01-1.06) : 1.11 (0.62-1.97) |
| Hartrampf et al. (2024) | New metastasis | : 1.02 (0.45-2.54) | NA |  | Change in PSMA-TV | : 1.001 (0.9972-1.004) |
| Eisazadeh et al. (2024) | NA |  | NA |  | highest SULmax-to-P-SUVmean (2.4) | : 4.00 (1.10-15.00) |
| Wang et al. (2023) | Visceral metastasis | : 9.90 (2.29-41.67) | PSA ≥96 | : 1.002 (1.000-1.003) | NA |  |
| Vanwelkenhuyzen et al. (2023) | NA |  | NA |  | NA |  |
| Thaiss et al. (2023) | VISION Criteria Visceral metastasis Liver metastasis | : 1.01 (0.5-2) : 1.31 (0.6-2.7) : 1.58 (0.7-3.5) | Hb ≥10 PSA ≥116 | : 0.37 (0.20-0.70) : 2.11 (1.10-4.10) | NA |  |
| Telli et al. (2023) | Moderate-severe pain PSA DT (≦2.4mo) | : 10.88 (2.90-40.80) : 15.7 (3.70-66.40) | Hb | : 0.59 (0.41-0.83) | FDG > PSMA | : 4.90 (1.19-20.62) |
| Tauber et al. (2023) | Time from diagnosis to 177LuPSMA <9yr Previous chemotherapy | : 1.7 (0.9-3.5)  : 2.0 (1.0-4) | Hb <11.9 LDH >253 | : 2.2 (1.1-4.4) : 2.3 (1.1-5.0) | NA |  |
| Stangl-Kremser et al. (2023) | NA |  | NRL CALGB risk group CTC count | : 1.05 (1.00-1.11) : 2.69 (1.82-4.69) : 2.92 (1.70-4.46) | NA |  |
| Şahin et al. (2023) | ECOG PS≥2 | : 1.92 (1.01-3.65) | Alb ≥3.4 NLR ≥2.7 dNLR ≥1.67 MLR ≥0.41 PLR ≥134.27 SII ≥570.39 | : 0.36 (0.16-0.82) : 3.32 (1.66-6.65) : 1.34 (1.08-1.67) : 2.53 (1.35-4.76) : 2.47 (1.23-4.96) : 2.17 (1.09-4.32) | NA |  |
| Pathmanandavel et al. (2023) | NA |  | NA |  | Increase in PSMA TTV Increase in 18F-FDG TTV Increase in 18F-FDG SUVmax | : 5.1 (1.5-17.1) : 1.04 (0.4-2.9) : 1.3 (0.4-4.5) : 1.8 (0.5-6) |
| Kim et al. (2023) Handke et al. (2023) | NA |  | NA |  | SUVmax50 SUV≥3 | : 1.0035 (1.0014-1.0054) : 1.0013 (1.0007-1.0019) |
| Karimzadeh et al. (2023) | Age Pretreatments ≥2 Previous 223Ra Previous chemotherapy Bone metastasis Visceral metastasis | : 1 (1-1) : 1.3 (0.8-2) : 1.1 (0.7-1.7) : 1.1 (0.7-1.7) : 2.5 (1-5) : 1.5 (1-2.1) | ALP LDH Hb PSA ALP increase (≥20%) LDH increase (≥20%) Hb decrease (≥20%) | : 1.0 (1.0-1.1) : 1.0 (1.0-1.1) : 1.0 (1.0-1.0) : 1.0 (1.0-1.0) : 1.0 (1.0-1.1) : 1.2 (1.0-1.3) : 1 (0.8-1.4) | High scintigraphic uptake | : 0.8 (0.6-1.1) |
| John et al. (2023) | NA |  | NA |  | Δ SPECT TTV  Δ SUVmax Δ SUVmean SUVmean <7 | : 0.85 (0.3-2.2) : 0.76 (0.2-2.6) : 1.3 (0.3-6) : 2.4 (0.9-6.1) |
| Hotta et al. (2023) | Previous DOC Number of metastasis≥20 Pelvic nodal metastasis Distant nodal metastasis Bone metastasis Liver metastasis | : 1.5 (1.02-2.21) : 1.1 (0.74-1.65)  : 0.94 (0.68-1.31) : 1.52 (1.07-2.17) : 1.35 (0.7-2.6) : 2.04 (1.37-3.02) | NA |  | PSMA tumor volume | : 1.00 (1.00-1.00) |
| Gaal et al. (2023) | Previous chemotherapy lines: one line Previous chemotherapy lines: two lines Brain metastasis | : 2.12 (1.18-3.83)  : 1.25 (0.60-2.60)  : 9.2 (3.03-27.90) | PSA De Ritis ratio | : 1.001 (1.000-1.002) : 1.27 (1.03-1.56) | NA |  |
| Cytawa et al. (2023) | NA |  | Hb CRP LDH ALP PSA | : 1.1 (0.80-1.50) : 1.99 (1.27-3.12) : 1.01 (1.00-1.01) : 1.00 (1.00-1.00) : 1.01 (1.00-1.02) | NA |  |
| Chua et al. (2023) | Number of cycles | : 0.68 (0.57-0.82) | PSA velocity ALP Hb | : 1.01 (1.004-1.012) : 1.001 (1-1.002) : 0.7 (0.57-0.86) | NA |  |
| Ballal et al. (2023) | NA |  | NA |  | Radiographic progression | : 8.264 (1.43-16.50) |
| Zarehparvar Moghadam et al. (2022) | Cumulative 177Lu-PSMA dose ≥12.95 | : 0.04 (0.01-0.26) | Hb ≥11.2 Plt ≥32.7×104 | : 0.12 (0.02-0.64) : 8.5 (1.77-40.74) | Bone superscan pattern | : 38.99 (2.37-641.08) |
| Wrenger et al. (2022) | Liver metastasis | : 6.98 (2.58-18.86) | Hb LDH | : 0.698 (0.56-0.87) : 1.073 (1.02-1.13) | NA |  |
| Kemppainen et al. (2022) | NA |  | NA |  | para-aortic MTV | : 1.51 (1.12-2.02) |
| Yadav et al. (2021) | Number of lines of chemotherapy Number of previous standard lines of treatment ≥2 Anti-cancer treatment after 177Lu-PSMA | : 1.5 (1.018-2.321)  : 2.9 (1.207-7.012)  : 0.3 (0.164-0.55) | NA |  | NA |  |
| Michalski et al. (2021) | ECOG PS Liver metastasis | : 4.5 (1.8-11.6) : 7.6 (1.2-49.3) | NA |  | FDG+/PSMA- | : 4.9 (1.7-14.3) |
| Kind et al. (2021) | NA |  | PSA | : 1.78 (1.03-3.09) | Tumor volume on PET | : 1.003 (1.001-1.006) |
| Gafita et al. (2021) | Time since diagnosis Previous chemotherapy Number of metastasis≥20 Bone metastasis Liver metastasis | : 0.92 (0.89-0.95) : 1.53 (1.01-2.37) : 1.66 (1.12-2.44)  : 1.10 (0.57-2.13) : 2.11 (1.38-3.23) | Hb | : 0.85 (0.77-0.95) | SUVmean | : 0.94 (0.9-0.98) |
| Calais et al. (2021) | Previous chemotherapy (0-1 vs. ≥2) ECOG PS≥2 Visceral metastasis Treatment activity (6.0 vs. 7.4Gy) | : 1.15 (0.55-2.37)  : 1.26 (0.54-2.92) : 1.94 (0.94-4) : 0.83 (0.4-1.75) | NA |  | NA |  |
| Ahmadzadehfar et al. (2021) | Previous chemotherapy ECOG PS 0 (Ref: 2) ECOG PS 1 (Ref: 2) Bone metastasis Liver metastasis | : 1.56 (1.20-2.03) : 0.33 (0.13-0.46) : 0.57 (0.42-0.77) : 3.70 (1.90-7.21) : 2.39 (1.82-3.15) | NA |  | NA |  |
| Bülbül et al. (2020) | Visceral metastasis | : 1.51 (0.68-3.35) | LDH ≥220 Hb ≥13 | : 1.61 (0.73-3.55) : 4.81 (1.08-21.43) | NA |  |
| Barber et al. (2019) | KPS (80) Time from diagnosis to 177LuPSMA (5yr) Cumulative 177Lu-PSMA dose (16) Bone metastasis Visceral metastasis Previous chemotherapy | : 1.83 (1.07-3.14) : 0.61 (0.38-0.97)  : 0.5 (0.31-0.81)  : 2.32 (0.81-6.69)  : 1.69 (1.02-2.8) : 1.53 (0.88-2.67) | PSA (60) Hb (7.5) ALP (220) | : 1.27 (0.73-2.21) : 2.06 (1.18-3.58) : 1.83 (1.08-3.1) | NA |  |

* PSA-PFS

**Supplementary Table 5.** Summary of prognostic factors for PFS in each included study

| Study | Clinical features | HR (95% CI) | Hematologic biomarkers | HR (95% CI) | Radiographic features | HR (95% CI) |
| --- | --- | --- | --- | --- | --- | --- |
| Slootbeek et al. (2024) | NA |  | NA |  | NA |  |
| Sathekge et al. (2024) | Previous Chemotherapy Previous ARPI Treatment with 177Lu-PSMA RLT Liver metastasis Peritoneal metastasis Number of cycles of 225Ac-PSMA RLT ECOG PS≧2 | : 1.37 (1.012-1.853) : 1.282 (0.966-1.702) : 1.1 (0.838-1.443)  : 1.878 (1.308-2.696) : 4.08 (1.675-9.937) : 0.97 (0.905-1.039)  : 1.031 (0.793-1.34) | Anemia | : 1.249 (0.965-1.615) | NA |  |
| Satapathy et al. (2024) | NA |  | NA |  | NA |  |
| Raychaudhuri et al. (2024) | Liver metastasis PARPi received Gene mutation  DDR  TSG | : 3.08 (1.34-7.09) : 1.44 (0.7-2.97)  : 0.75 (0.39-1.45) : 1.93 (1.05-3.54) | NA |  | PSMA SUVmean≧10 | : 0.59 (0.25-1.42) |
| Neubauer et al. (2024) | NA |  | NA |  | NA |  |
| Michalski et al. (2024) | NA |  | NA |  | NA |  |
| Kuo et al. (2024) Armstrong et al. (2024) | Bone metastasis Liver metastasis Lymph node metastasis | : 2.27 (1.30-4.00) : 1.56 (1.05-2.27) : 0.70 (0.51-0.95) | LDH | : 1.36 (1.17-1.59) | SUVmean(Whole body) SUVmean(Bone) SUVmax(Lymoh node) SUVmax(Soft tissue) | : 0.86 (0.82-0.9) : 0.87 (0.82-0.91) : 0.98 (0.98-0.99) : 0.98 (0.97-0.99) |
| Kinikoglu et al. (2024) | Age(≥65) Gleason Group (High/very high vs. Intermediate) Visceral metastasis Previous Chemotherapy 177-Lu line (≥3 vs. 2) Previous ARPI Combination therapy | : 1.56 (0.94-2.56) : 1.19 (0.65-2.17)  : 1.20 (0.74-2.00) : 1.41 (0.59-3.45) : 1.49 (0.85-2.63) : 0.38 (0.14-1.01) : 0.37 (0.21-0.64) | NA |  | NA |  |
| Kafka et al. (2024) | NA |  | GGT>31U/L | : 1.431 (0.91-2.26) | NA |  |
| Hein et al. (2024) | NA |  | NA |  | NA |  |
| Hartrampf et al. (2024) | NA |  | NA |  | NA |  |
| Eisazadeh et al. (2024) | NA |  | NA |  | NA |  |
| Wang et al. (2023) | NA |  | ALP ≥161 | : 1.006 (1.001-1.011)* | PSMA-vol ≥ 363 | : 1.05 (0.97-1.09) |
| Vanwelkenhuyzen et al. (2023) | ECOG PS≧2 Liver metastasis Prior lines | : 3.21 (1.23-8.40) : 0.4 (0.08-2.03) : 0.88 (0.61-1.26) | PSA ALP Hb LDH | : 1.00 (1.00-1.00) : 1.00 (1.00-1.00) : 0.87 (0.67-1.12) : 1.00 (1.00-1.00) | NA |  |
| Thaiss et al. (2023) | NA |  | NA |  | NA |  |
| Telli et al. (2023) | NA |  | CRP | : 1.40 (1.10-1.70)* | NA |  |
| Tauber et al. (2023) | Time from diagnosis to 177LuPSMA <9yr Previous chemotherapy | : 1.7 (1.0-3.0) : 1.7 (0.9-3.1) | Hb >11.9 LDH >253 | : 2.2 (1.2-3.9) : 1.8 (1-3.2) | NA |  |
| Stangl-Kremser et al. (2023) | NA |  | NA |  | NA |  |
| Şahin et al. (2023) | NA |  | NA |  | NA |  |
| Pathmanandavel et al. (2023) | NA |  | NA |  | NA |  |
| Kim et al. (2023) Handke et al. (2023) | NA |  | NA |  | NA |  |
| Karimzadeh et al. (2023) | NA |  | NA |  | NA |  |
| John et al. (2023) | NA |  | NA |  | Δ SPECT TTV  Δ SUVmax Δ SUVmean SUVmean <7 | : 1.9 (1.1-3.3)* : 1 (0.5-2.3)* : 1.2 (0.5-2.8)* : 2.4 (1.2-4.6)* |
| Hotta et al. (2023) | Previous DOC Number of metastasis≧20 Pelvic nodal metastasis Distant nodal metastasis Bone metastasis Liver metastasis | : 1.2 (0.83-1.73)* : 0.88 (0.6-1.28)* : 0.68 (0.5-0.94)* : 1.45 (1.04-2.04)* : 2.03 (1.07-3.85)* : 2.17 (1.46-3.21)* : 1.00 (1.00-1.00)* | NA |  | PSMA tumor volume | : 1.00 (1.00-1.00)* |
| Gaal et al. (2023) | NA |  | NA |  | NA |  |
| Cytawa et al. (2023) | NA |  | NA |  | NA |  |
| Chua et al. (2023) | NA |  | NA |  | NA |  |
| Ballal et al. (2023) | NA |  | NA |  | NA |  |
| Zarehparvar Moghadam et al. (2022) | NA |  | NA |  | NA |  |
| Wrenger et al. (2022) | NA |  | NA |  | NA |  |
| Kemppainen et al. (2022) | NA |  | NA |  | bone MTV | : 1.27 (1.03-1.55) |
| Yadav et al. (2021) | NA |  | NA |  | NA |  |
| Michalski et al. (2021) | NA |  | NA |  | NA |  |
| Kind et al. (2021) | NA |  | NA |  | NA |  |
| Gafita et al. (2021) | Time since diagnosis Previous chemotherapy Pelvic nodal metastasis Bone metastasis Liver metastasis | : 0.94 (0.92-0.97)* : 1.55 (1.03-3.00)* : 0.7 (0.51-0.97)* : 1.93 (1.07-3.52)* : 2.59 (1.69-3.95)* | NA |  | SUVmean | : 0.92 (0.88-0.96) |
| Calais et al. (2021) | Previous chemotherapy (0-1 vs. ≧2) ECOG PS≧2 Visceral metastasis Treatment activity (6.0 vs. 7.4Gy) | : 1 (0.4-2.52)  : 1.08 (0.55-2.12) : 1.79 (0.87-3.69) : 1.2 (0.58-2.49) | NA |  | NA |  |
| Ahmadzadehfar et al. (2021) | NA |  | NA |  | NA |  |
| Bülbül et al. (2020) | NA | NA | NA |  | NA |  |
| Barber et al. (2019) | Age (70) Time from diagnosis to 177LuPSMA (5yr) Bone metastasis Visceral metastasis Previous chemotherapy | : 1.07 (0.74-1.55) : 0.54 (0.37-0.78)  : 1.07 (0.61-1.87) : 1.46 (0.96-2.23) : 1.26 (0.84-1.89) | PSA (60) Hb (7.5) ALP (220) | : 1.09 (0.71-1.68) : 1.55 (1.01-2.37) : 2.13 (1.35-3.37) | NA |  |

* PSA-PFS

**Supplementary Table 6.** Summary of oncological outcomes based on PSA kinetics in each included study

|  | OS | | PFS | |
| --- | --- | --- | --- | --- |
| Study | PSA kinetics | HR (95% CI) | PSA kinetics | HR (95% CI) |
| Slootbeek et al. (2024) | NA |  | NA |  |
| Sathekge et al. (2024) | PSA decline of ≥50% | : 0.415 (0.307-0.561) | PSA decline of ≧50% | : 0.431 (0.33-0.565) |
| Satapathy et al. (2024) | PSA decline of ≥50% | : 0.36 (0.16-0.81) | NA |  |
| Raychaudhuri et al. (2024) | NA |  | NA |  |
| Neubauer et al. (2024) | PSA decline | : 0.32 (0.13-0.78) | NA |  |
| Michalski et al. (2024) | NA |  | NA |  |
| Kuo et al. (2024) Armstrong et al. (2024) | PSA decline of 0-50% PSA decline of ≥50%≦90% PSA decline of >90% | : 0.49 (0.36-0.66) : 0.30 (0.23-0.40) : 0.13 (0.08-0.20) | PSA decline of 0-50% PSA decline of ≧50%≦90% PSA decline of >90% | : 0.39 (0.26-0.58) : 0.28 (0.2-0.39) : 0.12 (0.07-0.2) |
| Kinikoglu et al. (2024) | NA |  | NA |  |
| Kafka et al. (2024) | NA |  | PSA decline of ≧30% after 1 cycle PSA decline of ≧30% after 2 cycle PSA decline of ≧30% after 3 cycle | : 0.54 (0.34-0.88)  : 0.50 (0.32-0.79)  : 1.17 (0.73-1.87) |
| Hein et al. (2024) | NA |  | NA |  |
| Hartrampf et al. (2024) | Change in PSA | : 1.004 (1.000-1.006) | NA |  |
| Eisazadeh et al. (2024) |  |  | NA |  |
| Wang et al. (2023) | NA |  | NA |  |
| Vanwelkenhuyzen et al. (2023) | NA |  | PSA decline of ≧50% | : 0.1 (0.04-0.30) |
| Thaiss et al. (2023) | NA |  | NA |  |
| Telli et al. (2023) | PSA decline of ≥30% after 1 cycle | : 0.98 (0.97-0.99) | NA |  |
| Tauber et al. (2023) | NA |  | NA |  |
| Stangl-Kremser et al. (2023) | NA |  | NA |  |
| Şahin et al. (2023) | NA |  | NA |  |
| Pathmanandavel et al. (2023) | PSA progression | : 3.5 (1.1-10.9) | NA |  |
| Kim et al. (2023) Handke et al. (2023) | NA |  | NA |  |
| Karimzadeh et al. (2023) | PSA increase (≥20%) | : 1.0 (1.0-1.0) | NA |  |
| John et al. (2023) | NA |  | NA |  |
| Hotta et al. (2023) | NA |  | NA |  |
| Gaal et al. (2023) | NA |  | NA |  |
| Cytawa et al. (2023) | PSA decline PSA progression | : 0.35 (0.11-1.01) : 1.98 (0.70-5.55) | NA |  |
| Chua et al. (2023) |  |  | NA |  |
| Ballal et al. (2023) | NA |  | PSA decline | : 0.15 (0.04-0.51) |
| Zarehparvar Moghadam et al. (2022) | NA |  | NA |  |
| Wrenger et al. (2022) | NA |  | NA |  |
| Kemppainen et al. (2022) | NA |  | NA |  |
| Yadav et al. (2021) | PSA decline of ≥50% | : 0.40 (0.25-0.59) | PSA decline of ≧50% | : 0.24 (0.15-0.39) |
| Michalski et al. (2021) | NA |  | NA |  |
| Kind et al. (2021) | NA |  | NA |  |
| Gafita et al. (2021) | NA |  | NA |  |
| Calais et al. (2021) | NA |  | NA |  |
| Ahmadzadehfar et al. (2021) | NA |  | NA |  |
| Bülbül et al. (2020) | PSA decline of ≥50% PSA decline | : 0.42 (0.16-1.14) : 0.59 (0.20-1.78) | NA |  |
| Barber et al. (2019) | NA |  | NA |  |

**Supplementary Table 7.** Summary of meta-analyses for OS

|  | Information of Included Studies | HR | 95% CI | p-value | Heterogeneity |
| --- | --- | --- | --- | --- | --- |
| OS |  |  |  |  |  |
| Clinical features |  |  |  |  |  |
| Age | 3 Studies n = 925 | 1.02 | 0.99-1.04 | 0.2 | I2 = 19.0%, Q = 1.2 (p =0.3) |
| ECOG ≥2 | 3 studies n = 655 | 1.99 | 1.45-2.74 | <0.001 | I2 = 0.0%, Q = 1.5 (p = 0.5) |
| GS | 2 studies n = 108 | 1.03 | 0.48-2.19 | >0.9 | I2 = 83.2%, Q = 6.0 (p = 0.01) |
| Visceral metastasis | 8 studies n = 816 | 1.65 | 1.33-2.05 | <0.001 | I2 = 0.0%, Q = 6.7 (p = 0.5) |
| Liver metastasis | 9 studies n = 2206 | 2.15 | 1.84-2.50 | <0.001 | I2 = 25.8%, Q = 10.8 (p = 0.2) |
| Bone metastasis | 6 studies n = 1868 | 2.09 | 1.39-3.13 | <0.001 | I2 = 45.6%, Q = 9.2 (p = 0.1) |
| Number of metastases≥20 | 2 studies n = 433 | 1.36 | 0.91-2.03 | 0.1 | I2 = 52.0%. Q = 2.1 (p = 0.1) |
| Time from diagnosis to PSMA-RLT | 2 studies n = 247 | 1.66 | 1.13-2.44 | 0.01 | I2 = 0.0%, Q = 0.01 (p = 0.9) |
| Pretreatment≥2 | 2 studies n = 422 | 1.77 | 0.82 | 3.81 | I2 = 60.2%, Q = 2.5 (p = 0.1) |
| Previous ARPIs | 2 studies n = 592 | 0.78 | 0.27-2.32 | 0.7 | I2 = 64.9%, Q = 2.9 (p = 0.09) |
| Previous Chemotherapy | 7 studies n = 1752 | 1.39 | 1.19-1.63 | <0.001 | I2 = 0.0%, Q = 4.8 (p = 0.6) |
| Cumlative PSMA-RLT dose | 2 studies n = 210 | 6.24 | 0.53 | 73.27 | I2 = 88.2%, Q = 8.5 (p < 0.01) |
| Radiographic progression | 2 studies n = 112 | 3.87 | 0.87-17.24 | 0.08 | I2 = 65.9%, Q = 2.9 (p = 0.09) |
| Hematologic features |  |  |  |  |  |
| PSA | 6 studies n = 1045 | 1.01 | 1.00-1.01 | 0.3 | I2 = 73.4%, Q = 11.3 (p = 0.01) |
| PSA (categorical) | 3 studies n = 283 | 1.26 | 0.84-1.89 | 0.3 | I2 = 64.5%, Q = 5.6 (p = 0.06) |
| Hb | 7 studies n = 827 | 1.25 | 1.09-1.43 | <0.01 | I2 = 61.5%, Q = 13.0 (p = 0.02) |
| Hb (categorical) | 5 studies n = 421 | 2.5 | 1.78-3.51 | <0.001 | I2 = 0.0%, Q = 3.3 (p = 0.5) |
| LDH | 5 studies n = 1017 | 1.16 | 0.96-1.39 | 0.1 | I2 = 89.3%, Q = 37.3 (p <0.001) |
| LDH (categorical) | 2 studies n = 125 | 1.94 | 1.12-3.35 | 0.02 | I2 = 0.0%, Q = 0.4 (p = 0.5) |
| ALP | 5 studies n = 1049 | 1.04 | 0.95-1.15 | 0.4 | I2 = 67.7%, Q = 9.3 (p = 0.03) |
| Radiographic features |  |  |  |  |  |
| SUVmean | 2 studies n = 744 | 0.91 | 0.85-0.97 | <0.01 | I2 = 79.6%, Q = 4.9 (p = 0.03) |
| SUVmean (categorical) | 2 studies n = 253 | 0.41 | 0.18-0.92 | 0.03 | I2 = 0.0%, Q = 0.0 (p > 0.9) |
| PSA kinetics |  |  |  |  |  |
| PSA decline | 3 studies n = 158 | 0.39 | 0.22-0.71 | <0.01 | I2 = 0.0%, Q = 0.8 (p = 0.7) |
| PSA decline ≥50% | 4 studies n = 694 | 0.41 | 0.32-0.51 | <0.001 | I2 = 0.0%, Q = 0.1 (p > 0.9) |
| PSA progression | 2 studies n = 96 | 2.56 | 1.19-5.51 | 0.02 | I2 = 0.0%, Q = 0.5 (p = 0.5) |

**Supplementary Table 8.** Summary of meta-analyses for PFS

|  | Information of Included Studies | HR | 95% CI | p-value | Heterogeneity |
| --- | --- | --- | --- | --- | --- |
| Clinical features | | | | | |
| Age (categorical) | 2 studies n = 271 | 1.24 | 0.86-1.79 | 0.2 | I2 = 29.8%, Q = 1.4 (p = 0.2) |
| ECOG ≥2 | 3 studies n = 588 | 1.34 | 0.75-2.38 | 0.3 | I2 = 60.0%, Q = 5.0 (p = 0.08) |
| Visceral metastasis | 3 studies n = 314 | 1.41 | 1.05-1.89 | 0.02 | I2 = 0.0%, Q = 0.8 (p = 0.7) |
| Liver metastasis | 4 studies n = 1222 | 1.75 | 1.37-2.25 | <0.001 | I2 = 44.9%, Q = 5.5 (p = 0.1) |
| Bone metastasis | 2 studies n = 718 | 1.56 | 0.74-3.26 | 0.2 | I2 = 71.1%, Q = 3.5 (p = 0.06) |
| Time from diagnosis to PSMA-RLT | 2 studies n = 247 | 1.8 | 1.32-2.45 | <0.001 | I2 = 0.0%, Q = 0.06 (p = 0.8) |
| Previous ARPI | 2 studies n = 592 | 0.77 | 0.24-2.49 | 0.7 | I2 = 81.4%, Q = 5.4 (p = 0.02) |
| Previous Chemotherapy | 4 studies n = 839 | 1.43 | 1.1-1.85 | <0.01 | I2 = 0.0%, Q = 0.4 (p = 0.8) |
| Hematologic features | | | | | |
| Hb (categorical) | 2 studies n = 247 | 1.75 | 1.24-2.47 | <0.01 | I2 = 0.0%, Q = 0.9 (p = 0.3) |
| PSA kinetics | | | | | |
| PSA decline ≥50% | 3 studies n = 666 | 0.25 | 0.12-0.52 | <0.001 | I2 = 80.9%, Q = 10.5 (p < 0.01) |

**Supplementary Table 9.** Summary of leave-one-out analysis

| Excluded study | HR | 95% CI | p-value | I2 | Cochran's Q test |
| --- | --- | --- | --- | --- | --- |
| OS_PSA |  |  |  |  |  |
| Michalski et al. (2024) | 1.01 | 1.00-1.01 | 0.3 | 73.4% | 11.3, p = 0.01 |
| Armstrong et al. (2024) | 1.00 | 1.00-1.01 | 0.3 | 72.7% | 7.3, p = 0.03 |
| Karimzadeh et al. (2023) | 1.01 | 1.00-1.01 | 0.3 | 73.4% | 11.3, p = 0.01 |
| Gaal et al. (2023) | 1.08 | 0.95-1.23 | 0.2 | 73.0% | 7.4, p = 0.02 |
| Cytawa et al. (2023) | 1.08 | 0.94-1.25 | 0.3 | 75.6% | 8.2, p = 0.02 |
| Kind et al. (2021) | 1.01 | 1.00-1.01 | 0.3 | 71.7% | 7.1, p = 0.03 |
| OS_Hb |  |  |  |  |  |
| Hein et al. (2024) | 1.29 | 1.09-1.53 | <0.01 | 64.2% | 11.2, p = 0.02 |
| Telli et al. (2023) | 1.21 | 1.06-1.37 | <0.01 | 57.4% | 9.4, p = 0.052 |
| Karimzadeh et al. (2023) | 1.25 | 1.09-1.43 | <0.01 | 61.5% | 13.0, p = 0.02 |
| Cytawa et al. (2023) | 1.29 | 1.13-1.47 | <0.001 | 58.2% | 9.6, p = 0.048 |
| Chua et al. (2023) | 1.22 | 1.04-1.42 | 0.01 | 61.0% | 10.3, p = 0.04 |
| Wrenger et al. (2022) | 1.22 | 1.04-1.42 | 0.01 | 62.2% | 10.6, p = 0.03 |
| Gafita et al. (2021) | 1.28 | 1.06-1.54 | 0.01 | 67.7% | 12.4, p = 0.01 |
| OS_LDH |  |  |  |  |  |
| Neubauer et al. (2024) | 1.10 | 0.95-1.27 | 0.2 | 89.3% | 27.9, p <0.001 |
| Armstrong et al. (2024) | 1.03 | 0.99-1.07 | 0.2 | 81.1% | 15.9, p <0.01 |
| Karimzadeh et al. (2023) | 1.34 | 0.90-1.98 | 0.2 | 91.9% | 37.1, p <0.001 |
| Cytawa et al. (2023) | 1.34 | 0.89-2.01 | 0.2 | 90.2% | 30.6, p <0.001 |
| Wrenger et al. (2022) | 1.34 | 0.86-2.09 | 0.2 | 90.4% | 31.1, p <0.001 |
| OS_ALP |  |  |  |  |  |
| Neubauer et al. (2024) | 1.05 | 0.95-1.15 | 0.4 | 77.6% | 8.9, p = 0.01 |
| Armstrong et al. (2024) | 1.00 | 1.00-1.00 | 0.1 | 0.0% | 0.4, p = 0.8 |
| Karimzadeh et al. (2023) | 1.07 | 0.91-1.26 | 0.4 | 78.5% | 9.3, p <0.01 |
| Cytawa et al. (2023) | 1.04 | 0.95-1.15 | 0.4 | 67.7% | 9.3, p = 0.03 |
| Chua et al. (2023) | 1.07 | 0.91-1.26 | 0.4 | 75.3% | 8.1, p = 0.02 |
| PFS_PSA decline ≥50% |  |  |  |  |  |
| Sathekge et al. (2024) | 0.18 | 0.08-0.41 | <0.001 | 59.4% | 2.5, p = 0.1 |
| Vanwelkenhuyzen et al. (2023) | 0.34 | 0.19-0.58 | <0.001 | 76.3% | 4.2, p = 0.04 |
| Yadav et al. (2021) | 0.23 | 0.05-0.94 | <0.001 | 86.7% | 7.5, p <0.01 |

**Supplementary Figure 1.** RoB2


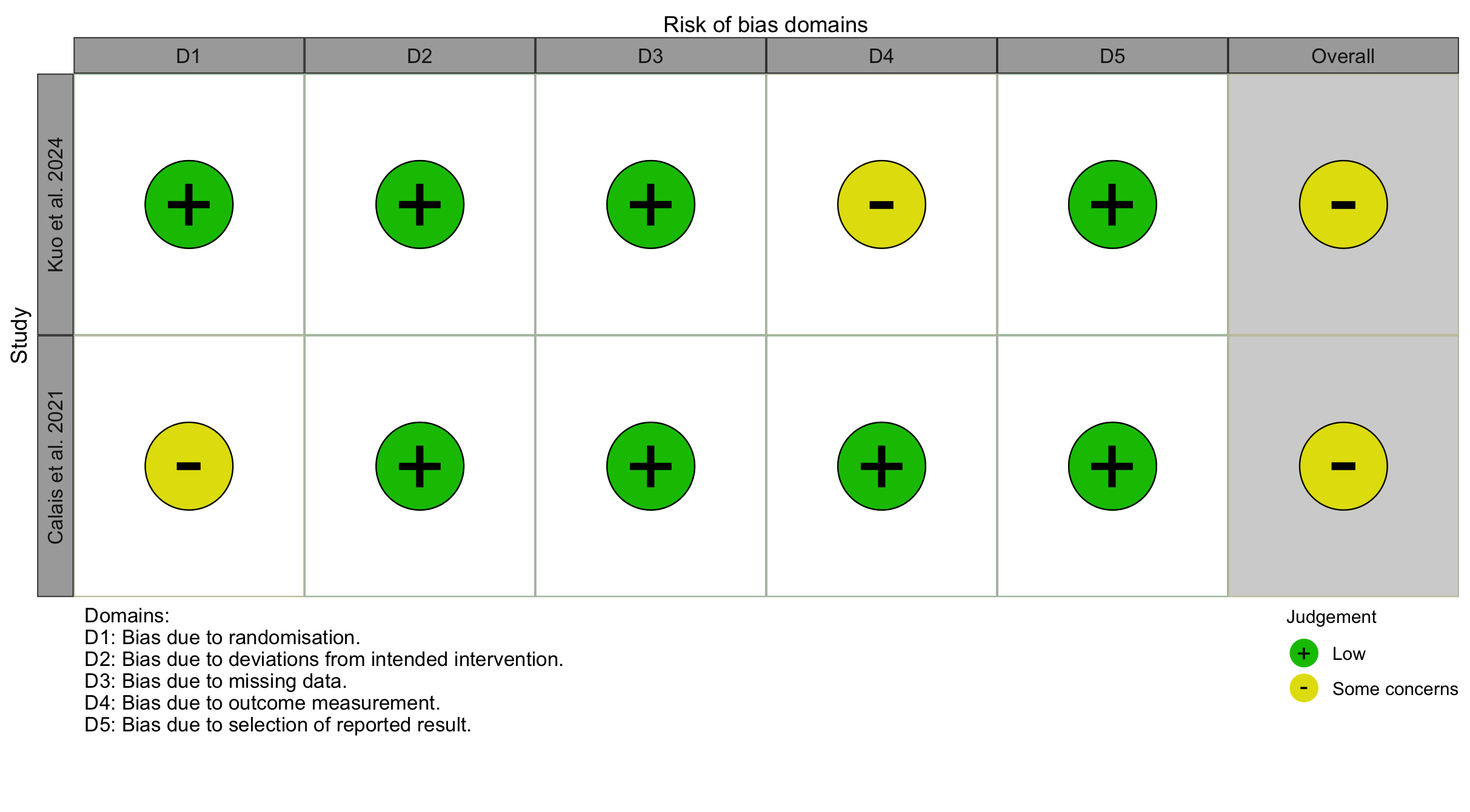


**Supplementary Figure 2.** Funnel plots of meta-analysis for OS

**Supplementary Figure 3.** Funnel plots of meta-analysis for PFS

**Supplementary Appendix 1**. Search strategy for systematic review and meta-analysis

Electronic searches were performed in the following databases to identify eligible studies

1. PubMed (n = 1,013)
2. EMBASE (n = 2,194)
3. Web of Science (n = 1,410)

There were no language or publication period limitations.

PubMed

#1 Search: prostatic neoplasms [MeSH Terms]

#2 Search: prostate cancer [Title/Abstract]

#3 Search: prostate carcinoma [Title/Abstract]

#4 Search: prostate neoplasm [Title/Abstract]

#5 Search: metastatic [Title/Abstract]

#6 Search: advanced [Title/Abstract]

#7 Search: PSMA [Title/Abstract]

#8 Search: prostate specific membrane antigen [Title/Abstract]

#9 Search: survival [Title/Abstract]

#10 Search: progression [Title/Abstract]

#11 Search: response [Title/Abstract]

#12 Search: (#1 OR #2 OR #3 OR #4) AND (#5 OR #6) AND (#7 OR #8) AND (#9 OR #10 OR #11)

EMBASE

('prostatic neoplasms':ab,ti OR 'prostate cancer':ab,ti OR 'prostate cancer':ti,ab) AND ('metastatic':ab,ti OR 'advanced':ab,ti) AND ('PSMA':ab,ti OR 'prostate specific membrane antigen':ab,ti) AND ('survival':ab,ti OR 'progression':ab,ti OR 'response':ab,ti)

Web of Science

#1 TS = (prostate cancer)

#2 TS = (prostate carcinoma)

#3 TS = (prostate neoplasm)

#4 TS = (metastatic)

#5 TS = (advanced)

#6 TS = (PSMA)

#7 TS = (prostate specific membrane antigen)

#8 TS = (survival)

#9 TS = (progression)

#10 TS = (response)

#11 TS = (#1 OR #2 OR #3) AND (#4 OR #5) AND (#6 OR #7) AND (#8 OR #9 OR #10)

#8 #1 AND (#2 OR #3 OR #4 OR #5) AND (#6 OR #7)

**Supplementary Appendix 2**. AMSTAR-2 checklist


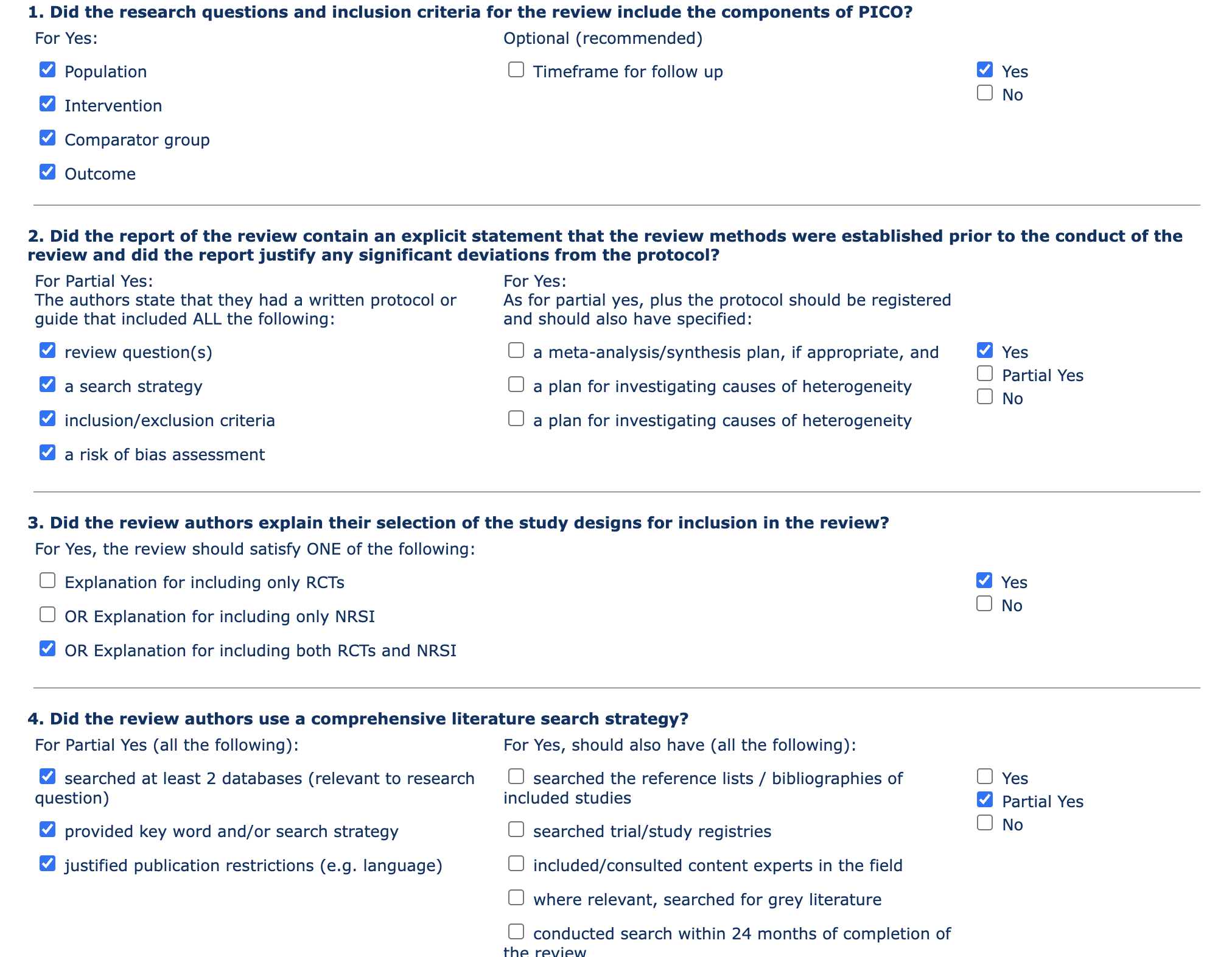


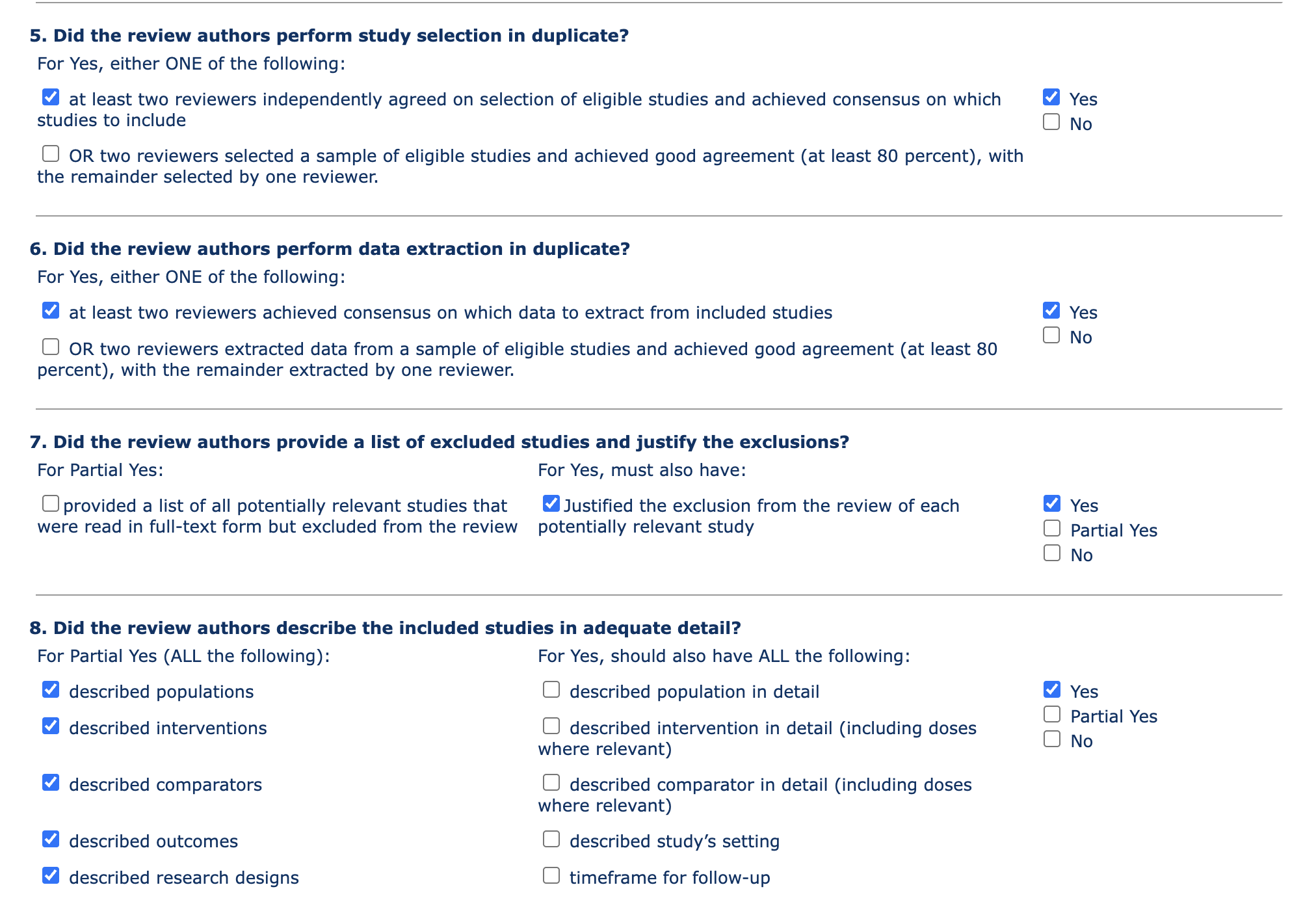


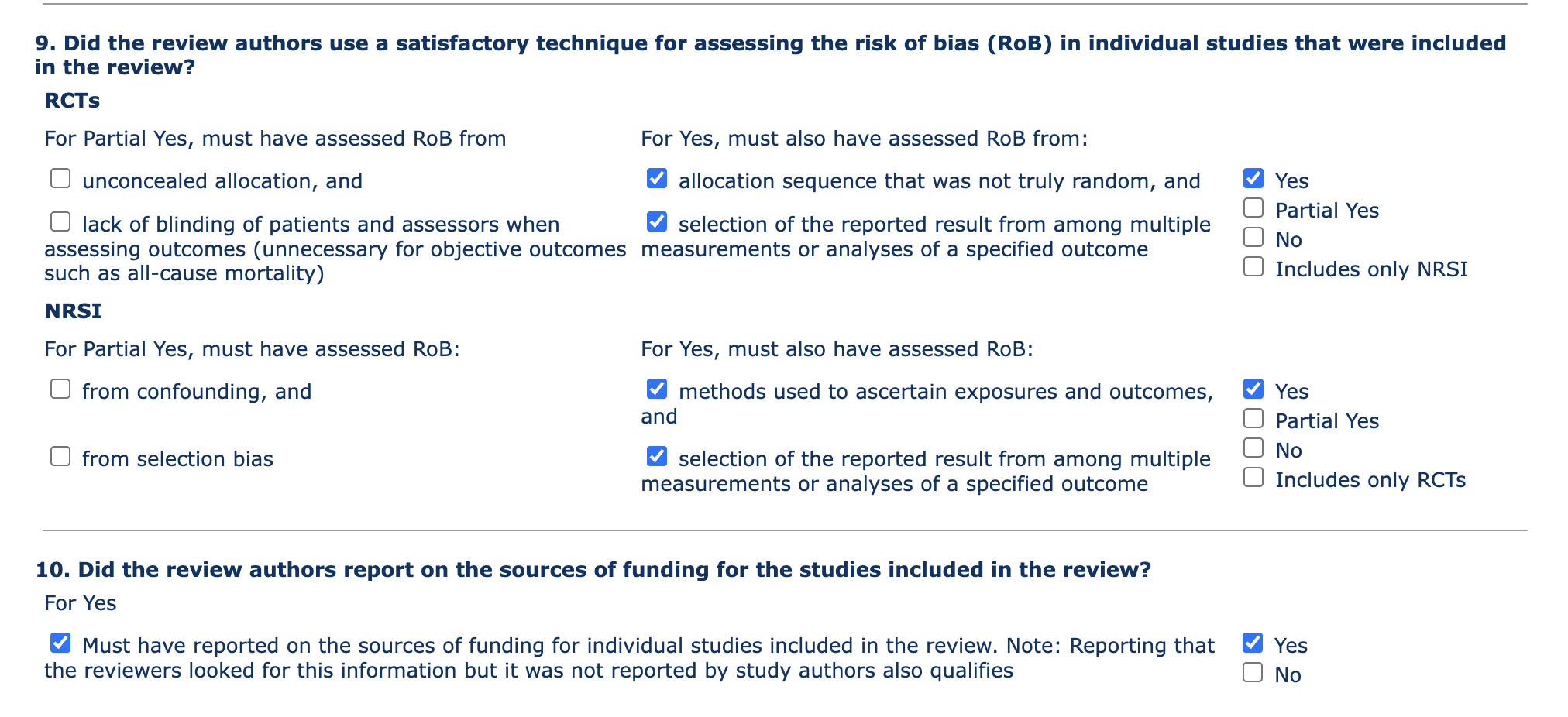


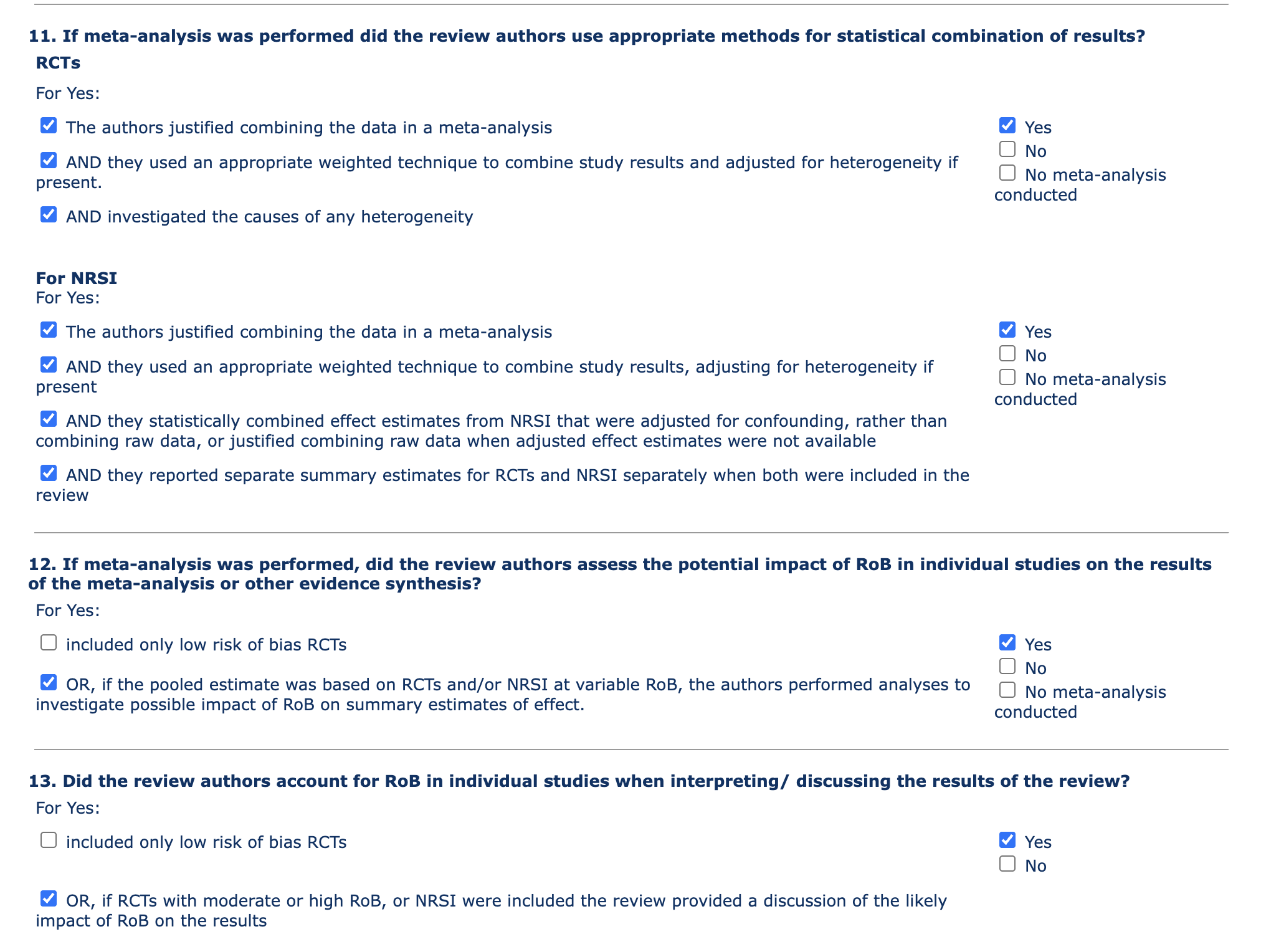


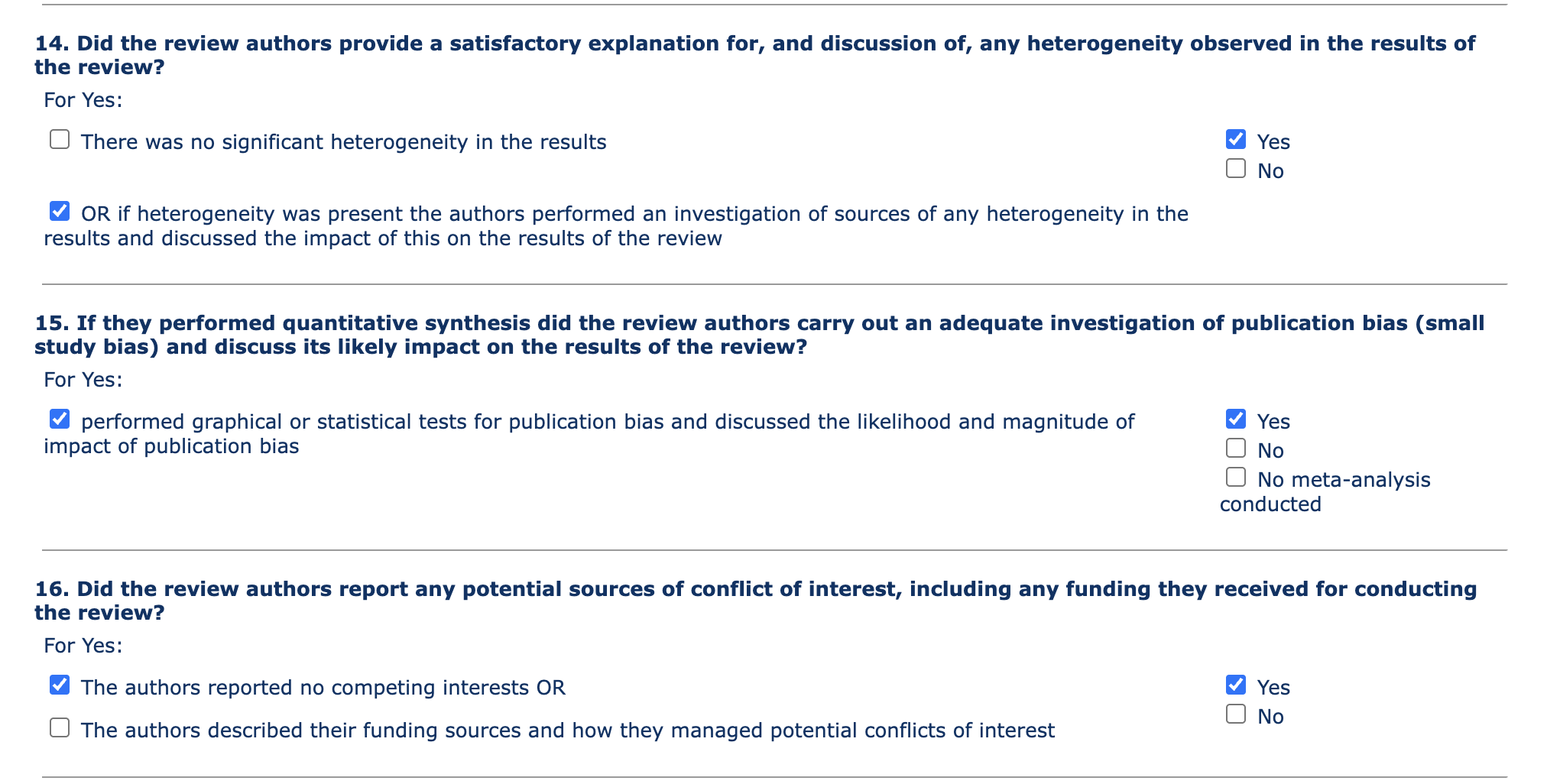

Supplement: Supplementary file 1 — Supplementary Information [file 41391_2025_1034_MOESM1_ESM.docx]
